# Immunoglobulin Heavy Chain Exclusion in the Shark

**DOI:** 10.1371/journal.pbio.0060157

**Published:** 2008-06-24

**Authors:** Karolina Malecek, Victor Lee, Wendy Feng, Jing Li Huang, Martin F Flajnik, Yuko Ohta, Ellen Hsu

**Affiliations:** 1 Department of Physiology and Pharmacology, State University of New York Health Science Center at Brooklyn, Brooklyn, New York, United States of America; 2 Department of Microbiology and Immunology, University of Maryland, Baltimore, Maryland, United States of America; Scripps Research Institute, United States of America

## Abstract

The adaptive immune system depends on specific antigen receptors, immunoglobulins (Ig) in B lymphocytes and T cell receptors (TCR) in T lymphocytes. Adaptive responses to immune challenge are based on the expression of a single species of antigen receptor per cell; and in B cells, this is mediated in part by allelic exclusion at the Ig heavy (H) chain locus. How allelic exclusion is regulated is unclear; we considered that sharks, the oldest vertebrates possessing the Ig/TCR-based immune system, would yield insights not previously approachable and reveal the primordial basis of the regulation of allelic exclusion. Sharks have an IgH locus organization consisting of 15–200 independently rearranging miniloci (V_H_-D1-D2-J_H_-Cμ), a gene organization that is considered ancestral to the tetrapod and bony fish IgH locus. We found that rearrangement takes place only within a minilocus, and the recombining gene segments are assembled simultaneously and randomly. Only one or few H chain genes were fully rearranged in each shark B cell, whereas the other loci retained their germline configuration. In contrast, most IgH were partially rearranged in every thymocyte (developing T cell) examined, but no IgH transcripts were detected. The distinction between B and T cells in their IgH configurations and transcription reveals a heretofore unsuspected chromatin state permissive for rearrangement in precursor lymphocytes, and suggests that controlled limitation of B cell lineage-specific factors mediate regulated rearrangement and allelic exclusion. This regulation may be shared by higher vertebrates in which additional mechanistic and regulatory elements have evolved with their structurally complex IgH locus.

## Author Summary

Lymphocytes provide a limitless repertoire of antigen receptors, but each lymphocyte expresses only one kind of receptor per cell in order to provide specific recognition and response to pathogen invasion. The restriction, called allelic exclusion, operates in tetrapod vertebrates from frogs to human beings. In mouse, immunoglobulin (Ig) heavy chain (H) exclusion depends on ordered activation of component parts of the highly complex, three-megabase IgH locus in a process that differentiates between the two alleles. However, the regulation and mechanisms ensuring allelic exclusion remain uncertain. Sharks represent the earliest vertebrates with an adaptive immune system; their IgH organization, consisting of multiple miniloci, is considered primitive and ancestral to the classical IgH locus in other vertebrates. We show that allelic exclusion nonetheless exists in shark B lymphocytes, although attained by alternative means. Thus, major aspects of the complex pathway described for allelic exclusion in mammals evolved with their IgH organization. Elucidating shared and divergent regulatory processes allows us to gain insight into the basis and evolution of allelic exclusion, which provides the foundation for the functioning of the adaptive immune system.

## Abstract 3

In B lymphocytes of most animals, only one allele is expressed at the antibody heavy-chain locus, while the other is shut down. Sharks have 15-200 such loci. How is antibody expression regulated in this early vertebrate?

## Introduction

The adaptive immune system in vertebrates is founded on lymphocytes expressing a vast, anticipatory repertoire of antigen receptors. Only a single species of immunoglobulin (B cells) or T cell receptor (T cells) is allowed per cell. This restriction is termed allelic exclusion, and it describes the requirements for monoallelic receptor gene expression in each cell (for a recent review, see [1]). Allelic exclusion is considered the basis of adaptive immune system function, but how this founding principle was established in evolution has been only a matter of conjecture [2]. In this report, we present data that clarify the basis of allelic exclusion in the shark, representative of the most primitive organism with an adaptive immune system shared by mice and human beings.

**Figure 1:**
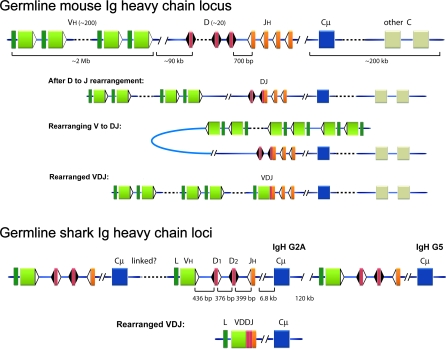
Comparison of Ig H Chain Genes in Mouse and Shark GL mouse Ig H chain locus: the mammalian H chain locus consists of a series of tandemly duplicated V_H_, D, and J_H_ gene segments that recombine during B cell development. The VDJ entity generated through the process “V(D)J rearrangement” is transcribed with one of the downstream constant (C) region genes, here simplified as single units (blue box is Cμ). The V_H_ is represented by olive boxes, preceded by the leader sequence in dark green, and flanked by the recombination signal sequence (RSS; white triangle) at the 3′ end, that consists of heptamer and nonamer motifs separated by a 23-bp spacer sequence. The RSS are the sites recognized by the RAG recombinase enzymes that mediate V(D)J rearrangement. As indicated, the distance between the 3′-most V_H_ and the first functional D is 90 kb [6]. The D gene segments, in red, are flanked on both sides by RSS (black triangles) containing 12-bp spacers, and the J_H_ gene segments (orange) with 23-bp spacer RSS. After D to J rearrangement: the first stage of rearrangement involves recombination between D and J_H_, with the intervening DNA excised. The DJ product is depicted as a fusion of the red and orange boxes, with the RSS flanking its 5′ end. Rearranging V to DJ: locus contraction and looping of the DNA allows distant V_H_ gene segments to approach and recombine with the DJ. The final VDJ product is shown as Rearranged VDJ. Germline shark Ig H chain loci: the IgM H chain genes in sharks and skates (cartilaginous fishes) are multiple “clusters” or miniloci, each consisting of V_H_, two D, one J_H_, and one Cμ gene (blue box). The gene segments in any nurse shark IgH gene are located about 400 bp apart as shown but are distant (e.g., 6.3–6.8 kb) from the Cμ1 exon. The physical relationships among the loci are not clear except for one instance, where the genes *G2A* and *G5* (both part of this study) were found linked and spaced 120 kb apart [23]. Rearranged VDJ: as demonstrated in this study, the four gene segments rearrange within the minilocus to VDDJ (called VDJ). In mouse IgH, gene rearrangement takes place in a strict order (D to J_H_ before V_H_ to DJ), but what the rearrangement process entailed in the shark miniloci was unknown up until this study.

The diversity of antigen receptors in lymphocytes is somatically generated by a recombination process in which various gene segments are joined together [3]. The number of gene segments and their organization vary amongst species but are generally comparable among all vertebrate classes, with the exception of the cartilaginous fishes, sharks and skates [4,5]. Figure 1 illustrates the differences between the complex mouse IgH locus and the multiple, minimalist shark clusters [4]. Elucidating their divergent and shared regulatory processes will allow us to understand the basis for allelic exclusion, the phenomenon that ensures specific recognition and response to pathogen invasion.

Each mammalian B lymphocyte must express an immunoglobulin (Ig) antigen receptor with a single specificity, although there are three loci that potentially encode two heavy (H) chains and four light (L) chains. The mouse IgH consists of an array of 200 V_H_ gene segments spaced over 2 Mb and located upstream from 10–13 D, four J_H_ gene segments, and eight constant (C) region genes [6] (Figure 1). Initiated by the RAG recombinase, the joining of V_H_, D, and J_H_ gene segments generates the ligand-binding V region that encodes the N-terminus of the H chain polypeptide [7]. Accessibility [8] of the gene segments to the recombinase is tissue-, developmental stage-, and gene-specific [9] and is associated with their transcription, although the nature of this connection is not entirely elucidated [10,11]. During B cell differentiation, chromatin domains encompassing the D, J, and Cμ genes become activated, probably through the intronic enhancer [12,13], allowing recombination of one of the D genes to a J gene segment. Only in B cells does V_H_ to DJ_H_ rearrangement take place to form VDJ, and this stage not only requires lineage-specific regulation, but sets in motion the process resulting in monoallelic H chain expression at the IgH locus. The chromatin domains encompassing the V_H_ become activated, and locus contraction is required to bring most of them into close proximity with the DJ [14,15]. After the completion of a productively rearranged VDJ, the H chain is expressed at the cell surface, and this initiates a feedback process by signaling the next step in differentiation [16,17]. Since the V to DJ step is asynchronous between the alleles, the first functional VDJ rearrangement will encode the antigen receptor.

Based on these mouse studies, a model for the regulation of allelic expression was developed. Recent work has shown that many factors at various levels—stage-specific expression of RAG [18], differentially activated chromatin domains [19], locus contraction and decontraction [14,20], and subnuclear relocation [15,21]—are involved. Because of the large distances between a rearranged DJ and the available V_H_ gene segments in animals such as mouse and humans, the locus contraction mechanism would appear to be part and parcel of the rearrangement process as well as its regulation. Moreover, the model is based on only one locus, that is, distinguishing one gene from its allele.

In contrast, IgM H chain in cartilaginous fishes (sharks and skates) is encoded by multiple (15–200), independently rearranging IgH loci (Figure 1). What is more, these miniloci may bypass the need for locus contraction, which seems to be a key regulatory step for monoallelic expression at a single IgH locus in the tetrapod model. The question of how allelic exclusion is managed in sharks has thus been a long-standing puzzle.

### Shark Immunoglobulin Gene Organization

In the nurse shark, Ginglymostoma cirratum, there are about 15 IgM H chain loci per genome, and every functional gene contains one V_H_, two D, and one J_H_ gene segments located within 2 kb ([22]; Figure 1, bottom). These miniloci are located at least 120 kb apart, and aside from two IgH genes depicted in Figure 1, their linkage relationships are not known [23]. Among outbred individuals there can be 9–12 active IgH, classified into subfamilies called Groups 1–5. A detailed characterization of two functional loci [22–24] and 78 of their rearrangements show that V(D)J recombination took place within the minilocus ([22,23] and V. Lee and E. Hsu, unpublished data). There do not seem to be long-distance recombination events between the widely separated IgH loci or, presumably, a major role for chromatin contraction in nurse shark IgH rearrangement.

To elucidate the rules for V(D)J recombination in the shark, we first investigated rearrangement patterns at the two defined shark H chain loci, asking whether differential V_H_, D, and J_H_ activation existed in the short (∼400 bp) intersegmental distances. We have found that all combinations are possible, and a completed VDJ is accomplished during one stage only, as if it were like the initial D to J step in mammals. Our results also confirmed that long-distance recombination between different IgH loci in B cells is rare, if it exists. Thus, two elements thought to be intrinsic to regulating the rearrangement process and resulting in allelic exclusion in mammals—ordered long distance recombination and chromatin contraction—are absent in sharks. Thus these findings tell us that certain mechanics of the rearrangement process can be dissociated from the phenomenon of allelic exclusion and that the two processes developed separately in evolution.

We investigated rearrangement in shark lymphocytes at the population and the single-cell level and established that H chain exclusion does occur in shark B cells, where only one or a few of its many IgH loci rearrange in any one cell. We also looked at IgH loci in shark thymocytes (precursor T cells, see Text S1). Although T cells do not express Ig, the IgH genes were extensively, although partially, rearranged; Ig transcripts were not detected. The differences between B cells and thymocytes demonstrated here suggest there exists in precursors to B and T cells an IgH chromatin state already permitting rearrangement, but in B cells it is further potentiated by lineage-specific factors, leading to efficient recombination at one or a few H chain genes and results in H chain exclusion. We propose that the molecular basis establishing allelic exclusion was achieved in the earliest vertebrates possessing Ig genes, and it is independent of the wide variation in Ig gene number observed in different species.

## Results

### Overview

**Figure 2:**
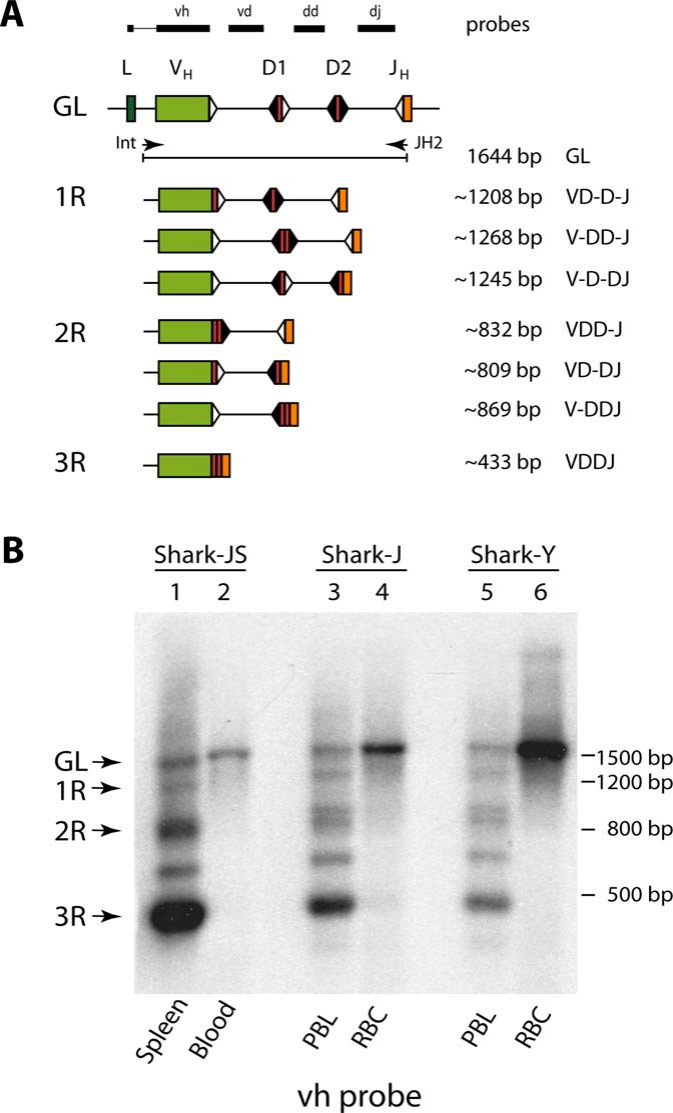
Somatic V(D)J Recombination Products in Lymphoid DNA (A) Anticipated rearrangement products detectable by PCR primers to shark IgH. The nurse shark Group 2 IgM H chain locus is depicted with V_H_, D1, D2, and J_H_ gene segments shown flanked by recombination signal sequences (see Figure 1, legend). Probes vh, vd, dd, and dj are shown above the regions from which they are derived. Expected configurations are illustrated and listed with approximate sizes of PCR products: 1R are VD-D-J, V-DD-J, or V-D-DJ (∼1,208–1,268 bp), 2R are VDD-J, VD-DJ, or V-DDJ (∼809–832 bp), and 3R are VD1D2J products (∼433 bp). The locations targeted by PCR primers Int and JH2 are shown as arrows. (B) GL and rearranged genomic Group 2 sequences from three individual sharks were amplified by PCR primers Int/JH2, specific for Group 2 sequences. Genomic DNA obtained from non-lymphoid tissue and lymphoid tissues were used to amplify genomic Group 2 sequences. Samples were electrophoresed on 1.5% agarose gel in Tris-borate-EDTA buffer, with molecular size markers on right (100-bp DNA ladder, NEB); the 1,644-bp GL sequence (arrow GL), is amplified from the RBC samples (lanes 2, 4, and 6). In contrast, a ladder of vh-hybridizing bands was obtained in lymphocytic samples (lanes 1, 3, and 5, as labeled). The 600-bp band was unclonable, probably single-stranded DNA.

The experiments are summarized as follows. We first focused on how rearrangement takes place in one IgH subfamily, Group 2, in tissues and isolated cell populations. We demonstrated that partially and fully rearranged V_H_ sequences can be amplified from lymphoid tissue DNA, but not from red blood cell (RBC) control DNA (Figure 2). All anticipated genomic rearrangement configurations were obtained (Table 1). These data demonstrated that rearrangement in sharks is different from the ordered, two-stage process observed in mammals.

**Table 1:**
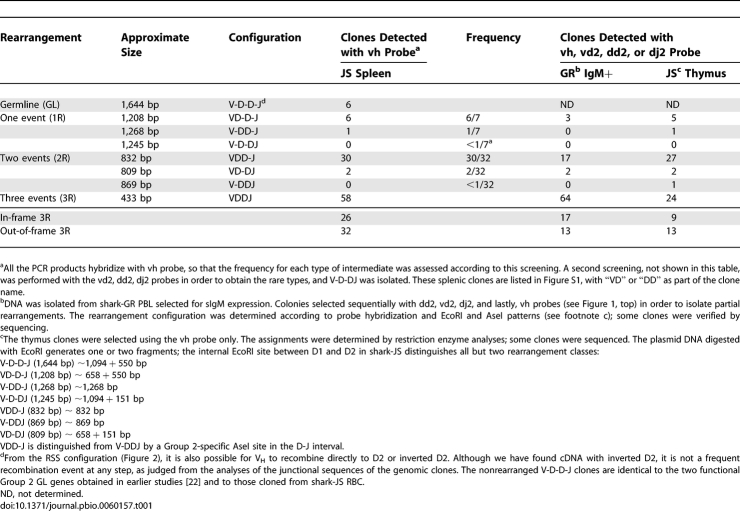
Genomic Group 2 VDJ Rearrangements in Shark Lymphoid Tissue

**Figure 3:**
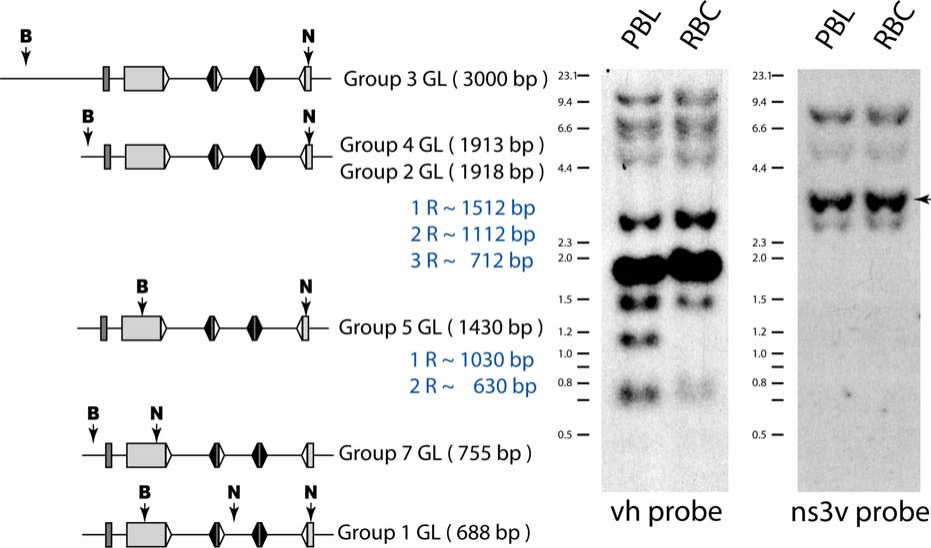
Detection of Rearranged DNA in PBL by Genomic Southern Blotting Left: the BamHI (B) and NcoI (N) sites in various IgM H chain loci are depicted, with the predicted size of vh-hybridizing fragment in parentheses. Groups 1–5 are functional genes (accession numbers: G1, DQ857386; G2; G3, DQ857384; G4A, DQ857390; G4C and G4G, DQ857388, G4E, DQ857387; and G5, DQ857385). The predicted sizes of some 1R-3R fragments after rearrangement are immediately below (in blue). In both GL and rearranged configurations, these genes will resolve as DNA bands from approximately 3 kb to 630 bp and smaller after the double digestion. Group 7 is a pseudogene, as are Group 6 and 8 [23,24], whose vh-hybridizing fragments form the higher bands at greater than 4 kb. Rearrangements, if any, in the pseudogenes have not been observed by genomic Southern blotting. Center and right: Southern blot analyses of genomic DNA from shark-J PBL and RBC digested with BamHI and NcoI. Molecular size markers are shown on the left (λ Hind III and 100-bp DNA ladder, NEB). The filter was hybridized with vh probe (center) after standardization with the ns3v probe (right). Band intensities were measured by phosphorimaging, followed by autoradiography with X-ray film. The 3.7-kb nonrearranging NS3 V region band (arrow) was used for standardizing transferred DNA (RBC lane = 1, PBL lane = 0.98) and verified by a comparison of TdT bands (RBC = 1, PBL = 1 [unpublished data]). The relative intensity of the 1.9-kb band in the vh probe panel was calculated as follows: (PBL vh probe score divided by 0.98) divided by RBC vh probe score. Using this calculation, the 1.9-kb PBL band is 77% of the 1.9-kb RBC GL band.

**Figure 4:**
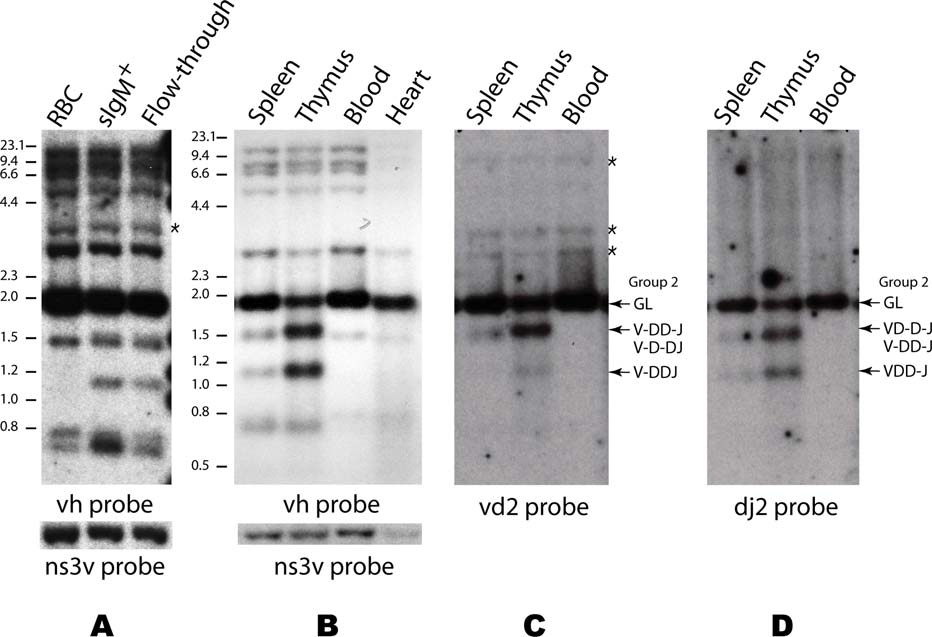
Detection of Rearranged IgH in Various Lymphoid Tissues (A) IgH rearrangement in sIgM+ cells as detected by the vh probe. DNA was extracted from shark-GR RBC, sIgM+ cells (B cells), and the depleted “flow-through” cells, digested with BamHI/NcoI, and subjected to Southern analysis. Evidence for T cell depletion of the sIgM+ population is shown in Figure S2. The blot in (A) was hybridized with the ns3v probe (3.7-kb band, bottom, as labeled) for standardization and rehybridized with the vh probe (top): RBC = 1.0, sIgM+ = 0.82, flow-through = 0.67. The calculated 1.9-kb vh signal: sIgM+ = 81% of RBC, flow-through = 92% of RBC. Asterisked bands in top (A) are remaining ns3v signals, all of which are greater than 3 kb. (B) IgH rearrangement in thymus and spleen as detected by vh probe. Genomic DNA from shark-JS spleen, thymus, blood, and heart were digested with BamHI/NcoI and treated as described above. The blot was hybridized with vh probe (top) and with ns3v (3.7-kb band, bottom). The 3.7-kb ns3v signal was used as standard: RBC = 1.0, spleen = 1.1, and thymus = 0.98. The 1.9-kb vh signals, calculated as described in Figure 3 legend, are spleen = 71% of RBC, thymus = 40% of RBC. (C) IgH rearrangement in thymus and spleen as detected by Group 2-specific vd2 probe. The Group 2 configurations are shown at the right. The vd2 probe detects the V-D intersegmental region of GL Group 2 genes, as well as those in rearranged configurations that have not recombined V to D (i.e., 1R, V-DD-J, and V-D-DJ; 2R V-DDJ). The filter was not stripped after hybridization with the ns3v probe; the asterisked bands are leftover ns3v signals. (D) IgH rearrangement in thymus and spleen as detected by Group 2-specific dj2 probe. The vd2-probed blot was stripped and hybridized to the dj2 probe. The Group 2 configurations are shown at right. The dj2 probe detects the D-J intersegmental region of GL Group 2 genes and those rearranged Group 2 genes that have not recombined D to J (i.e., 1R, VD-D-J and V-DD-J; 2R VDD-J).

**Figure 5:**
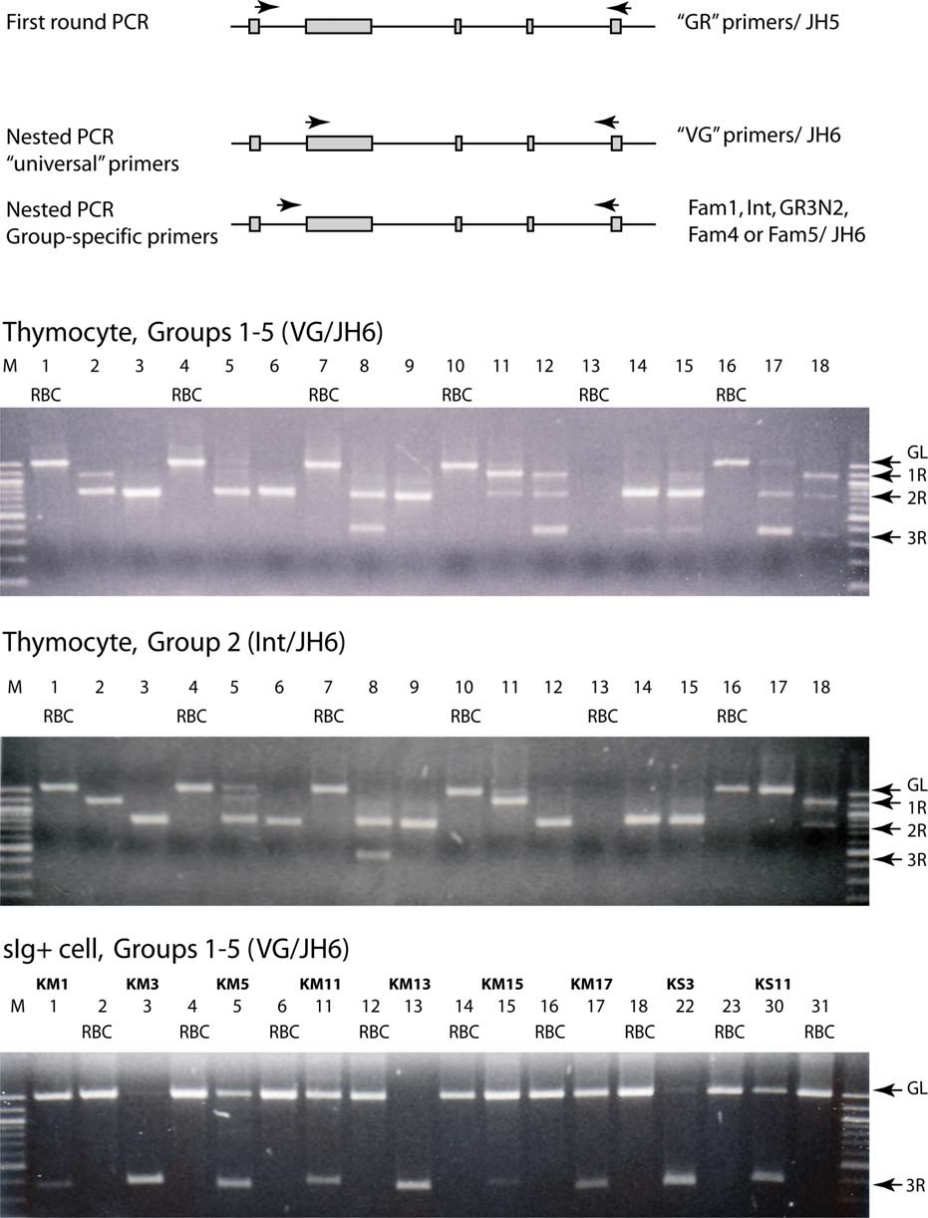
Single-Cell PCR of Nurse Shark Lymphocytes Diagram: A first PCR round was performed with degenerate primers targeting the leader intron (“GR” series) and J_H_ (JH5) sequences of Group 1–5 genes. Aliquots from the first round were amplified in a second round of PCR employing nested degenerate primers in V_H_ (“VG” series) and in J_H_ (JH6) that also collectively targeted the same genes. To identify the rearranged genes amplified by the VG/JH6 primers, separate second PCR rounds were done with JH6 in combination with five nested primers (Fam1, Int, GR3N2, Fam4, and Fam5) targeting leader intron sequence downstream of GR and specific to each of the five Groups. Top and middle: nurse shark thymocytes depleted of surface L chain-positive cells from shark-PI were picked by hand. After every second thymocyte, a RBC was picked as a check for the purity of isolation and processing. Top: A first PCR round was performed with the GR/JH5 primers targeting all Groups 1–5 genes; the nested round of PCR with VG/JH6 is shown in this panel. The expected band sizes are: 1.6 kb (GL), 1.2 kb (one rearrangement, 1R), 0.8 kb (2R), 0.4 kb (3R). Middle: one of the Group-specific nested reactions (primer pair Int/JH6), that targeting Group 2 genes, is shown in the middle. The DNA fragments of nested Group-specific PCR are expected to be overall about 52 bp longer than those described for the nested VG/JH6 reactions. Rearranged Group 2 products are identified in Figure S4. Bottom: surface L chain-positive peripheral blood leukocytes (i.e., B cells) from shark-GR were picked alternating with RBC for purity controls. PCR reactants and conditions are identical to that described in top panel. Each sIg+ PBL is flanked by a RBC. The names of the cells are shown per lane and correspond to those in Table 3.

Lymphoid tissue can carry B cells, which express IgM, and T cells, which do not. The initial experiments revealed the unexpected finding that somatic IgH recombination is also present in the thymus, an almost exclusively T cell–containing tissue. We further investigated this finding, using genomic Southern analyses. Compared to RBC and heart DNA, new—somatically rearranged—bands were observed in all lymphoid tissue DNA tested. These new bands were demonstrated to be V(D)J rearrangements mapping to predictable locations (Figure 3). The recombined bands were identified by probes detecting all V_H_ or only the Group 2 subfamily. A comparison of surface IgM-positive (sIgM+) B cell DNA with thymus DNA showed that their respective patterns differed in the quantity and quality of the rearranged bands (Figure 4). Thus, we discovered that V(D)J recombination is much more extensive but mostly incomplete in T cells. This result was confirmed through investigating the status of all functional IgH loci in thymocytes and in B cells by single-cell genomic PCR (Figure 5).

**Table 3:**
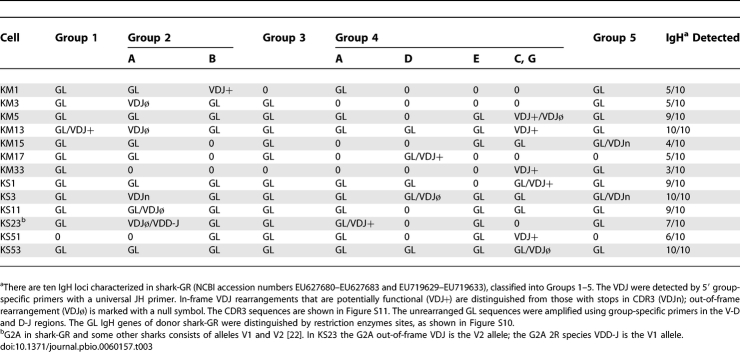
IgH Rearrangement in Single B Cells

**Figure 6:**
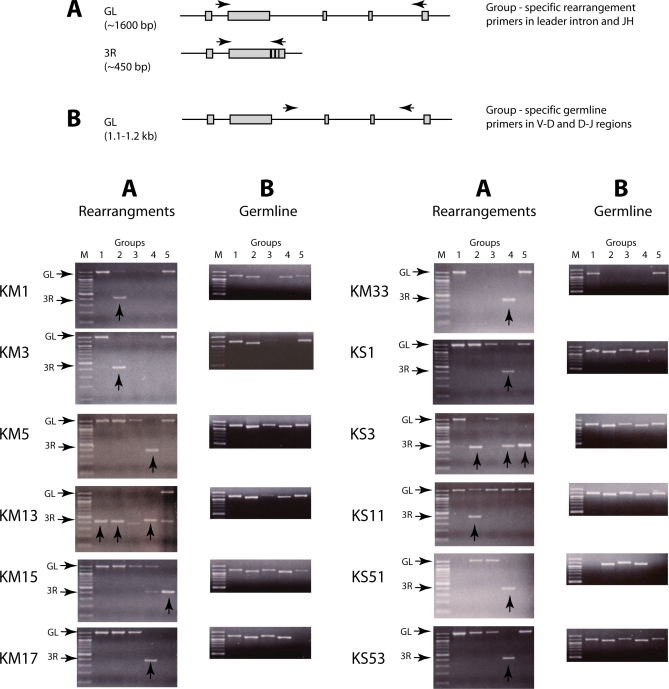
V(D)J Rearrangement in Single B Cells Single sIg+ B cells that contained 3R DNA (some shown in Figure 5, bottom) were selected. To ascertain the rearrangement status of all IgH in a cell, the first-round PCR sample was subjected to nested PCR reactions with Group-specific primers, five sets for “A” and five sets for “B” (diagram). Top: the A series (Fam1, Int, GR3N2, Fam4, or Fam5 with JH6) detecting rearrangement are shown with the locations they target in GL and recombined (3R) configuration. The B series (G1DF/G1JR, FD2/RD2, G3DF/G3JR, G4DF/DR34, and G5DF/G5JR) detect only nonrearranged IgH and are shown with the locations targeted in the V-D and D-J intersegmental regions. Bottom: the A rearrangement panels show that 1–3 VDJ (arrow) can be detected in each cell. Each 3R band was cloned and subject to the analyses detailed in Figure S9 and S11. The B panels show the IgH remaining in GL configuration. The method of identifying the bands, as well as individual members of the Group 2 and Group 4 subfamilies, is detailed in Figure S10. The summary of the identification of the GL and VDJ genes in each B cell is in Table 3.

**Figure 7:**
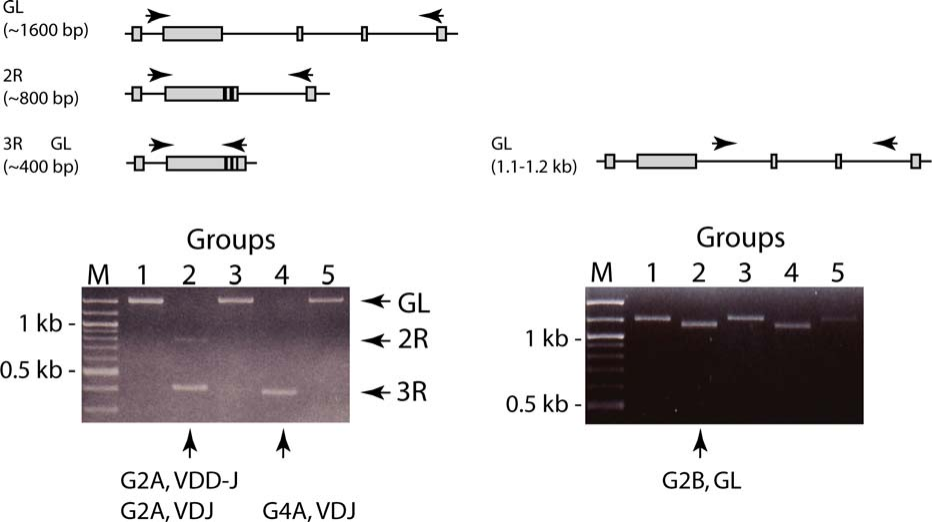
Example of an Incompletely Rearranged IgH in a Single B Cell, KS23 Group-specific PCR primers detected two 3R and one 2R band in the B cell KS23, as shown in the left panel. The Group 2 and Group 4 samples were cloned; the latter consisted of an in-frame VDJ and the former an out-of-frame VDJ involving allele V2 and a VDD-J (2R) with allele V1 of the *G2A* V_H_ gene [22]. The Group 2 subfamily contains two members, *G2A* and *G2B*. In the panel at the right, a nonrearranged Group 2 product found by the GL primers. This was, as expected, the *G2B* sequence which is distinguished from *G2A* by restriction enzyme analyses (supporting information Figure S10).

As predicted from the genomic Southern analysis results, only a few rearrangements could be obtained from single B cells, and these were fully rearranged VDJ; the other IgH loci were in the nonrearranged, or germline (GL), configuration (Figure 6 and Table 3). Unlike in thymocytes, partial rearrangements (Figure 7) were infrequent in B cells. These results show that in the developing B cell, there was a limited number of genes activated to rearrange, but once initiated, the recombination process went efficiently to completion.

**Figure 8:**
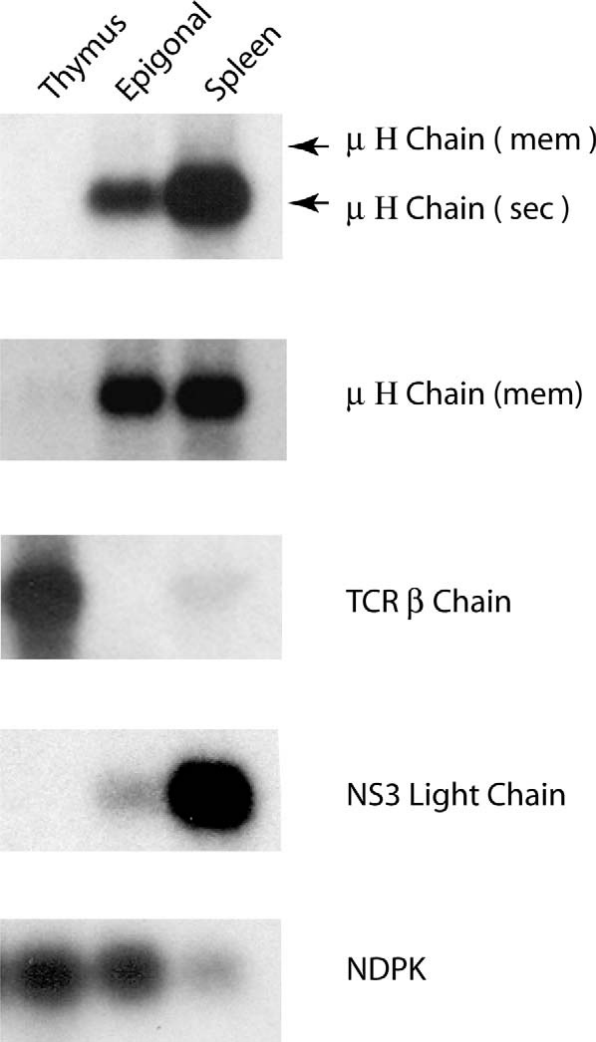
RNA Transcripts Present in Shark Lymphoid Organs Northern analyses were performed with total RNA (ca. up to 5 μg) from shark-JS thymus, epigonal organ, and spleen, and the blot hybridized with probes containing the sequences of: domains Cμ3–Cμ4, the transmembrane sequence of Cμ, TCRβ C region, NS3 C region and nucleotide diphosphate kinase (NDPK). Probes are described in Materials and Methods. These detected, respectively, transcripts of both the μ H chain secreted and membrane form, μ H chain membrane form alone, TCR β chain, NS3 L chain, and NDPK. There is considerably more of the secreted form of H chain than the membrane form, and an arrow shows the relative position of the latter transcript. Signal patterns and intensities from a second L chain probe, ns4c (not shown) were identical to those detected by ns3c. Signal intensities from vh probe (unpublished data) were similar to results from Cμ membrane probe, mem.

In contrast, multiple and mostly incomplete Ig rearrangements were found in single thymocytes (Table 2), and neither Ig H chain transcripts nor L chain expression and rearrangement could be detected in the thymus (Figure 8). This ability of DNA to act as substrate for RAG in the absence of transcription suggests a previously unknown state of chromatin activation. It was possible to detect this state only in an animal with multiple, independently rearranging sites, but such an observation signals that RAG may act on nontranscribing loci in other organisms as well. We propose that IgH in all shark precursor lymphocytes can be acted upon by RAG recombinase but that B lineage-specific factors are responsible for regulated rearrangement—and H chain exclusion—in the B cells.

**Table 2:**
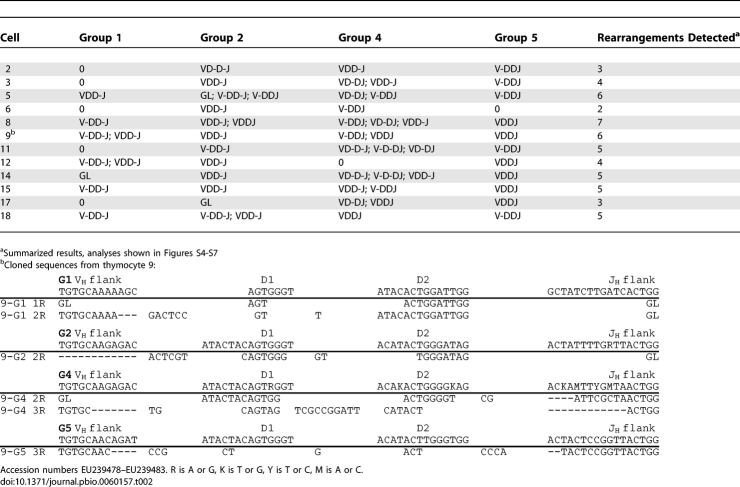
Presence of Multiple IgH Rearrangements in Single Thymocytes

### V(D)J Rearrangements Amplified from Lymphoid Genomic DNA

PCR primers (Int/JH2; Figure 2) targeting the leader intron of Group 2 V_H_ and the J_H_ gene segment amplified DNA sequences of 1.6 kb from an individual shark (-JS) whole blood DNA (Figure 2B, lane 2); this band contained the two functional Group 2 genes in the nonrearranged, or GL, configuration (see PCR and probes, [22]). The Int/JH2 primers amplified the same 1.6-kb fragment from erythrocyte DNA in other genetically unrelated individuals (Figure 2B, lanes 4 and 6), demonstrating that Group 2 GL gene segments are in the organizational configuration depicted in Figure 1.

The intersegmental distances in Group 2 as well as other IgM genes are all about 400 bp (Figure 1). A single, initial somatic rearrangement event (1R), such as joining of V to D1, would delete this interval and reduce the total V to J genomic span detected by the Int/JH2 primers to about 1,200 bp. Likewise, two rearrangement events (2R) would give rise to a PCR product of about 800 bp, and three rearrangements (3R) 400 bp.

From lymphoid (spleen and peripheral blood leukocytes [PBL]) genomic DNA, a ladder of PCR products hybridizing to the vh probe (Figure 2) can be detected, corresponding to the anticipated sizes of partial and completed genomic rearrangements (Figure 2B, lanes 1, 3, and 5). The arrows at the left of lane 1 point to the fragments that were later cloned from all three sharks and identified as having one, two, or three rearrangements. Splenic lymphocytes and PBL from adult sharks do not express RAG recombinase ([25]; W. Feng and E. Hsu, unpublished data), so that these rearrangement intermediates would be relics from earlier stages of lymphocyte differentiation.

### No Strict Order in the Joining of Gene Segments

PCR products obtained from shark-JS splenic DNA were cloned, and the insert sequences are classified by size in Table 1. Within each size group, different rearrangement combinations were found, but some are more frequent than others. The junctions (Figure S1) show that each clone is unique, with the typical diversity generated by trimming and N/P region addition.

In order to determine which cells contained these rearrangements, surface IgM-expressing (sIgM+) cells were isolated from PBL (see Figure S2). The PCR reactions performed on this population and the thymus both amplified sequences that showed higher frequency of V_H_ to D1 joining (Table 1), but all combinations exist. The fully rearranged VDJ (i.e., VDDJ) from the sIgM+ cells tended to be in-frame, whereas in those from the spleen and thymus, the nonfunctional ones are in the majority (last two rows, Table 1). Thus, it appeared that IgH recombination had occurred in both precursor T and B lymphocytes. The pattern of rearrangement for Group 2, as demonstrated by the frequencies of the intermediate configurations, was similar in all samples.

Although the 5′ primer is specific for Group 2, the 3′ primer could target any IgH J_H_. If rearrangement occurred between Group 2 V_H_ and another locus, it would have been possible to detect non-Group 2 intersegmental sequence in the partial configurations shown in Table 1. (All but one of the partially recombined clones had rearranged within Group 2 loci, as ascertained by sequencing or restriction enzyme analyses. In one thymus VDD-J clone, the V_H_ originated from Group 2, but the D2, D2-J intersegmental sequence and J_H_ were from Group 1; only four nucleotides belong to the D1 of either Group. If this sequence were a PCR artifact, the area of homology would have to have been in the N region sequence between V_H_ and D1 or D1 and D2. We screened a total of 58 thymic VDD-J [unpublished data], but this was the only apparent instance of interlocus rearrangement outside these Group 2 IgH.)

Two kinds of probes were used during screening, all of which had been derived from a GL Group 2 bacteriophage clone (Materials and Methods): the vh probe and the intersegmental probes, vd2, dd2, and dj2 (Figure 2, top). The latter were used to detect the infrequent recombination intermediates in these experiments (Table 1, footnote a; e.g., the Group 2 V-D-DJ configuration is vd2+, dd2+, dj2−). On genomic Southern blots, they proved to be specific for Group 2 only, whereas in contrast, the vh probe cross-hybridizes with nurse shark V gene segments from all subfamilies (see Figure S3). In the following genomic Southern blotting experiments, these probes were used to detect rearrangement globally (vh probe) as well as specifically at three Group 2 IgH (vd2, dj2 probe) in lymphoid tissue DNA.

### IgH Rearrangement Visible by Genomic Southern Blotting

All the genes encoding nurse shark IgM H chain have been cloned and the functional genes can be classified into five subfamilies, Groups 1–5 (see legend, Figure 3); the V_H_ gene segments share >75% nucleotide identity [24]. The various vh-hybridizing bands in RBC DNA can be correlated with anticipated fragment sizes after BamHI/NcoI digestion (Figure 3, RBC lane in vh panel with map). Although the DNA amounts are similar in RBC and PBL lanes (Figure 3, right, ns3v panel), there are novel vh bands in the PBL sample (Figure 3, PBL lane in vh panel). Compared to RBC DNA, the bands at 1,500 bp and 700 bp in PBL are more intense, and a new band appears at 1,100 bp. These three bands correspond to predicted configurations of rearranged DNA from the various IgH, but mostly from Groups 2 and 4 (Figure 3, 1R-3R in blue). At the same time, the 1.9-kb band encompassing the Groups 2+4 GL gene segments in PBL is ca. 23% less than that of the RBC counterpart (see Figure 3, legend), demonstrating loss of the GL band after acquisition of rearranged configurations.

### Heavy Chain Loci Rearranged in Thymic Tissue

We observed that the relative amount of DNA rearranged was different between thymus (predominantly T cell) and sIgM+ cells (B cells from PBL). Although the images in Figure 4 are from X-ray films, phosphorimager analyses were performed for a quantitative analysis. We centered our analyses on depletion of the 1.9-kb band because it is a single GL configuration of known genes Group 2+4, whereas “gain” measurements cannot be so clearly resolved. For instance, gain of signal in the 1.5-kb region means a combination of Group 2/4 1R plus nonrearranged GL Group 5, but minus an unknown amount of loss by Group 5 rearrangement.

To obtain a rough idea of the proportion of rearranged IgH in B cells only, the DNA from sIgM+ cells from shark-GR PBL was compared to DNA from its RBC (Figure 4A). The “flow-through” sample is from the population mostly depleted of sIgM+ cells and consists of thrombocytes, granulocytes, and lymphocytes (T cells and some B cells that slipped through). An obvious difference between sIgM+ and the flow-through population is the greater intensity of the 700-bp band in the former (Figure 4A). This band mostly contains 3R species, suggesting that most Group 2+4 rearrangements in B cells are VDDJ.

There is a 19% signal reduction of the 1.9-kb Group 2+4 GL band in the sIgM+ lane. The “flow-through” DNA also contained few rearrangements, as assessed by both loss of GL (8%) and gain of rearranged bands. However, unlike the sIgM+ sample, the “flow-through” was a mixture of cell types, and lymphocytes in PBL can range from 5%–30%.

DNA from shark-JS spleen, thymus, whole blood, and heart were compared. There were rearranged Ig bands in spleen and thymus, but these were not detected in blood or heart DNA (Figure 4B). The frequency of lymphocytes in whole blood is very low (0.02%–0.12%, 1 PBL/250 RBC), and in shark-JS, the heart tissue was bled out. The amounts of DNA in the first three lanes in Figure 4B are similar, and a comparison of the intensities of the 1,500-bp, 1,100-bp, and 700-bp bands between the spleen and thymus samples in Figure 4B suggests that more Ig rearrangements were present in the thymus DNA. Indeed, upon calculation, 60% of the thymus vh-hybridizing GL 1.9-kb Group 2+4 band was depleted.

In summary, in one B cell-enriched sample (sIgM+ cells from PBL), 19% of Group 2+4 genes were rearranged and mostly to VDDJ, whereas in thymus, 60% of Group 2+4 genes were rearranged, mostly to intermediate configurations.

In order to analyze these blotted DNA samples in more detail, we performed hybridizations with probes that detect only Group 2 IgH (*G2A*, *G2B*, and pseudogene *G2C*). The resulting bands can be correlated with Group 2 rearrangement intermediates characterized in Table 1 (Figures 4C, vd2, and 4D, dj2). The 1,500-bp (VD-D-J/V-DD-J) and 1,100-bp (VDD-J) bands detected by dj2 probe in thymus appear to be as intense as the 1.9-kb Group 2 GL signal and reflect the high frequency of these events (Table 1). Again, using the GL band as an internal reference, the Group 2 vd2 signal demonstrates that other Group 2 configurations (V-DD-J/V-D-DJ at 1,500 bp and V-DDJ at 1,100 bp) do exist but are less frequent, consistent with results from Table 1.

### Single-Cell PCR

All the previous experiments were performed on mixed and purified cell populations, and although we can anticipate the general trend in T cells (many and partial rearrangements) and in B cells (few and completed rearrangements), this remains to be shown at the individual cell level. Single-cell analysis was made possible by previous studies in which all the GL IgH sequences in nurse shark have been characterized [22,23] so that degenerate, universal primers could be synthesized, targeting and detecting only the functional genes. Likewise, it was possible to devise primers specific for each Group, just as Int was specific for Group 2 genes. We first focused on thymocytes. We picked single thymocytes, performed single-cell PCR with the universal primers in a two-stage assay (Materials and Methods) and demonstrated the existence of multiple IgH rearrangements. For controls, an erythrocyte was picked after every two thymocytes. Out of 24 RBC, five failed to amplify and the remaining 19 showed only the GL bands. Of the 48 thymocytes, 44 had a variety of 1R, 2R, and 3R bands. Figure 5 (top) shows the results from the first 18 cells after the second round of PCR with nested universal primers. Other nested PCR was also performed with Group-specific primers to Group 2 (Figure 5, middle panel, and Figure S4), Group 1 (Figure S5), Group 4 (Figure S6), and Group 5 (Figure S7). The summary of these results is shown in Table 2. For the most part, little GL sequence can be detected, except in the RBC controls, suggesting either that most of the IgH had rearranged or that the many rearrangements caused the longer GL fragments to be out-competed. Either possibility is the result of widespread IgH rearrangement in the single thymocyte. The various anticipated rearrangements could be cloned from any thymocyte (Table 2, footnote).

The thymocyte result is in contrast to what we obtained in B cells (Figure 5, top and bottom, respectively). Using the identical PCR conditions and reagents, the PCR performed with the universal primers on surface L chain–positive B cells produced predominantly 3R bands. Moreover, GL bands were also present in almost every one of these samples. When several samples of B cell 3R fragments were analyzed on denaturing gels, they appeared to consist of only one or two species per sample (Figure S8).

### Heavy Chain Exclusion in Shark B Cells

We went on to identify the rearranged and nonrearranged IgH in single B cells. Using the Group-specific primers, we performed nested PCR on the first-round products of the single B cells (Figure 6, A amplifications) and found that each B cell carried one or only a few rearrangements. Each 3R band was cloned and the number of VDJ species determined per cell (detailed in Figure S9 and legend). We also amplified nonrearranged GL sequence from each cell by using Group-specific primers directed to the intersegmental regions (Figure 6, B amplifications) and identified the genes in each fragment by restriction enzyme sites. These tests were tailored for the donor, shark-GR, all of whose IgH were isolated and sequenced for this experiment (Figure S10). Table 3 summarizes the results from 13 B cells. The CDR3 sequences of these VDJ are shown in Figure S11; the rearrangements in Table 3 are indicated as out-of-frame (VDJø) or in-frame (VDJ+) or nonfunctional (in-frame but containing stops, VDJn). All 13 B cells contained 3R rearrangements, and one cell (KS23) carried a 2R species as well (Figure 7).

We have shown a remarkable disparity between T cells and B cells in IgH gene configuration. In thymocytes, there are multiple and mostly partial IgH rearrangements per cell. Although we cannot claim to detect every VDJ rearrangement present in a B cell, the many IgH genes that remain in GL configuration support the observation that few IgH were rearranged in a single B cell. Many VDJ in Table 3 are out-of-frame or contain stops, consistent with there being only one functional VDJ per cell.

In one cell, KM13, we found two VDJ that were both in-frame (*G1*; *G4CG*) and carried no stops in CDR3 (Table 3), whereas the third VDJ is out-of-frame (*G2A*). One of the former (*G4CG*) encodes a CDR3 of 24 amino acids, an aberration among nurse shark cDNA CDR3, which range from 4–17 codons (average 11.6 codons, *n* = 64) in one study [24] and 7–16 codons in another (adult *G4* cDNA, average 11.3 codons, *n* = 41, W. Feng and E. Hsu, unpublished data). However, the *G1* VDJ not only contains a CDR3 of average size (11 codons) but is also the only one that has been hypermutated, and its mutations show evidence of positive selection (National Center for Biotechnology Information [NCBI; http://www.ncbi.nlm.nih.gov/] accession number EU719628). There are eight substitutions, with only those in the CDRs resulting in replacement changes. Three point mutation changes in FR2 and FR3 are synonymous, but the CDR1 point mutation (R to W), and the point mutation (Q to K) and 3-bp tandem mutation (S to R) in CDR2 all result in nonconserved changes. Tandem mutations are characteristic of the nurse shark hypermutation process [22], and the frequency of PCR-induced changes after 70 cycles in these studies is 0.14% (13/10,371 bp), or less than one change per 400-bp VDJ fragment. We do not know whether the VDJ with the 24-codon CDR3 encodes an IgM protein, but it is clearly not part of the selection process acting on this hypermutating B cell.

Perhaps, considering their very different CDR3 sizes, there is L chain preference for one polypeptide enabling its expression. We then ask, how often do two rearrangements result in similar CDR3? There are four cells (KM5, KM13, KS3, and KS23) in which more than one VDJ is present although most of these are nonfunctional. The junction sizes range widely. The number of nucleotides between TGT in the V_H_ flank and TGG in the J_H_ flank are 34 bp/45 bp in the KM5 VDJ, 39 bp/44 bp/78 bp in KM13, 24 bp/59 bp/72 bp in KS3, and 33 bp/41 bp in KS23 (Figure S11). With six flanks trimmed and three sites for N region addition per VDJ, it seems unlikely that any two VDJ in a B cell, even if both are potentially functional, would have such similar CDR3 sequence content and loop sizes that they would combine equally well with the available L chain. Thus, constraints operating at two levels—the combination of the random nature of V(D)J rearrangement and L chain compatibility—serve to enforce H chain exclusion.

We propose that rearrangement ceases with the production of a successful H and L chain combination. There are few partially rearranged IgH present in B cells, as the 2R in KS23. Here, the constellation of in-frame (presumed functional) *G4* VDJ, the out-of-frame *G2A* VDJ, and the partially rearranged *G2A* 2R allele suggest that there was a signal for cessation of rearrangement for the *G2A* in VDD-J configuration once a viable μ protein was generated.

### Rearranged IgH Transcribed Only in B Cells

Ig transcripts from functional and nonfunctional rearrangements can be cloned from B cell-containing shark-JS lymphoid tissue using Int/JH2; we found that the use of a primer in leader intron selects for Ig transcripts unspliced in this region, the majority of which are from aberrant (out-of-frame, partially rearranged) genes. The 3R (VDDJ) sequences were obtained from spleen cDNA, and many were mutated regardless of whether they were productive VDJ or not. Of the 17 2R events we cloned, two were VD-DJ, and one of them carried several mutations in the V region although not in the D-D intergenic sequence. Of 15 independent VDD-J clones, nine were mutants, of which seven contained substitutions throughout V and the D-J intergenic sequence. The mutation patterns are typical of the type previously described in shark Ig, consisting of point and tandem mutations [26]. One such example, A36, is shown in Figure S12.

In contrast, there is very little Ig mRNA in shark-JS thymus, as observed by northern blotting (Figure 8), whereas these and other probes for nurse shark L chain isotypes detect abundant mRNA in spleen and epigonal organ. TCR β chain is abundant in thymus RNA. Reverse transcriptase PCR (RT-PCR) experiments using Int/JH2 to detect Group 2 2R in thymus cDNA were negative (unpublished data). Given the extent of thymic IgH rearrangement described in the preceding section, we conclude that if Ig transcription does occur in precursor T cells, the RNA species are at extremely low levels.

### Light Chain Rearrangement in Spleen and Thymus

As the IgH rearrangements in thymus were a surprising observation, we investigated whether Ig L chain genes were also active in any way. The nurse shark L chains are encoded by three isotypes, NS3, NS4, and NS5 [27]. NS4 is most abundant (about 60 to 70 IgL), consists of both rearranging and germline-joined loci, and contributes about 90% of the L chain cDNA clones; neither NS4 nor the germline-joined NS3 could be detected in thymus RNA (Figure 8). In NS5, there are four genes, two of which can rearrange; they each consist of one V_L_ and one J_L_ gene segment and one C exon. Whereas somatically rearranged NS5 genomic sequences can be amplified from any source that contains B cells, none was observed in the shark-JS thymus DNA sample (Figure S13). The rearranged NS5 band in the control spleen sample was visually apparent in ethidium gels.

In thymus, few if any NS5 genes somatically rearrange, and certainly not on the scale of the IgH. Thus, like in mouse, Ig rearrangements in thymocytes involve only the H chain loci.

## Discussion

The mechanisms that contribute to generating H chain exclusion—differential chromatin domain activation, locus contraction—have evolved with and are a consequence of the complex mammalian Ig organization. In this study, we have shown that these processes are not necessary to effect H chain exclusion in all vertebrates. Our model, the nurse shark, provides a naturally minimalist IgH locus with four rearranging gene segments. Because rearrangement can be initiated by any gene segment pair, it seems unlikely that the spatially close V, D, and J elements are regulated separately from each other or subject to different chromatin accessibility constraints. Preliminary data from non-Group 2 subfamilies show that rearrangement patterns can vary considerably; for instance, in Group 5, V-DDJ is a prominent configuration that is rarely observed for Group 2 (Tables 1 and 2). Such observations suggest that, once the gene is accessible to recombinase, a preferred order of rearrangement is probably governed by locus-specific factors, for instance, the relative recombination efficiency of particular RSS pairs.

With one possible exception, the 97 1R/2R rearrangements isolated in this study (Table 1) occurred within the minilocus, supporting conclusions drawn from cDNA observations. Long-distance recombination events and sequential chromatin activation do not occur during the shark IgH V(D)J recombination process, demonstrating that in the absence of major aspects of the complex pathways described for mouse allelic exclusion, H chain exclusion will still be managed by limitation of rearrangement.

We established in this report that IgM receptors appear to be clonally expressed in nurse shark and likely all elasmobranch fishes. In one study in the clearnose skate [28] one to three different CDR3 μ junctions were obtained by RT-PCR from single cells. Unfortunately, most of the 100–200 clearnose skate IgH are not characterized, and a number of them are germline-joined VDJ, which make these results difficult to evaluate. We have classified all nurse shark Ig H chain genes in a BAC library and determined those that are functional [23]. Our PCR primers target these genes only. We found ten functional H chain genes in the individual shark-GR and detected in its B lymphocytes one to three VDJ rearrangements per cell. At best, only one VDJ per cell was potentially functional. The other IgH were nonproductive VDJ or in GL configuration.

We believe that most elasmobranch B cells express one dominant H chain mRNA and one IgM receptor. Eason and coworkers [28] hypothesized that one gene is activated at a time, like in the multigene olfactory receptor system. A mechanistic connection seems unlikely, in the absence of an evolutionary relationship between genes encoding Ig superfamily and seven transmembrane domain proteins. From our studies, it appears that either a few IgH loci are rearranging at the same time in the pro-B cell or there are sequential “tries” before a viable H chain protein is generated. The answer is possibly in between.

Partially rearranged 1R and 2R configurations do exist in B cells as best demonstrated by cloning of mutated cDNA (Figure S12) and the 2R species detected in B cell KS23 (Figure 7 and Table 3). The relic incomplete rearrangement configurations in B cells might suggest a feedback mechanism that functions with staggered initiation of rearrangement among loci. Alternatively, a few IgH are fully activated to rearrange, more or less simultaneously; hence the infrequent laggard 2R in the population. In such a scenario, there would be a limited but clear possibility for allelic inclusion. That we do not find many such examples suggests that the probability for two viable rearrangements is low, and as illustrated in the case of KM13, L chain preference DNA might permit only one H chain polypeptide for the receptor. As in mammals, ongoing shark IgH rearrangement probably ceases with the formation of a functional VDJ and expression of the IgM receptor. If L chain rearrangement occurs subsequently, the H chain loci might be transiently inactivated, as occurs with the non-expressed allele in mouse [20]. We have speculated that H and L chain rearrangement occur simultaneously in shark [29], and all rearrangement ceases with the formation of a viable cell surface receptor. However, there currently is no experimental evidence favoring either possibility.

The question remains, how are 15 or 100 IgH loci to be regulated if more than one gene can be activated per cell?

In point of fact, genetically manipulated model systems with more than two H chain genes have been studied. In interspecies hybrid tetraploid and triploid *Xenopus* [30] and in mice triallelic for IgH [31] allelic exclusion of H chain was observed, despite the increased number of potentially competing genes. There is no reason to believe that in these animals Ig expression is regulated any differently than their diploid version. If that is the case, H chain exclusion is initiated by nonsynchronously occurring rearrangement, and it does not matter how many available genes there are. It is generally accepted that the crucial step differentiating two alleles or multiple genes should be at V to DJ stage [32]. However, in the shark, there is no such second stage; the asynchrony must occur at the initiating step of rearrangement.

Liang and coworkers [33] inserted a GFP reporter into the kappa locus to mark its activation and found that the gene was transcribed at an unexpectedly low frequency in pre-B cells. They suggested that allelic exclusion at the kappa locus is based on probabilistic enhancer activation. Possibly a predetermined allele preference [34,35] contributes to the initial choice, but it was also suggested [33] that a competition for transcription factors would forestall activation of the second gene.

We observed few but mostly fully recombined IgH per shark B cell and propose that there are limiting amounts of trans-factors that target a gene for highly efficient, processive rearrangement, such that however many IgH genes are in the genome, recombination in B cells does not commence at the same time at more than one location. The focused activity at a few IgH also may have the effect of draining other components from general use. Since shark IgH genes lack the usually well-conserved upstream octamer motif [22,36], their trans-factors must differ from and are not competed for by L chain genes if they rearrange at the same time. The first compatible and viable H and L chain combination forming a receptor will generate the feedback signal. If by chance more than one viable H chain is produced at the same time, they may be differentiated by their ability to pair with the available L chain.

The surprising finding in these studies is V(D)J recombination at multiple IgH loci in every thymocyte, and despite the numerous H chain rearrangements present, Ig transcripts are not detected. The majority of thymic IgH are left incomplete as 1R or 2R, further underlining the difference of their estate from that in B cells. Since these IgH genes are not transcribed as in B cells, despite the extensive rearrangement, and are mostly not fully recombined, essential components are obviously lacking in thymocytes. Taken altogether, we propose that in those thymocytes which are in the process of actively recombining their TCR genes also harbor IgH in a rearrangement-permissive state, and this is possibly a prelude to full activation of the chromatin, which can only be achieved in the presence of B lineage-specific components that would include IgH transcription factors. Since cDNAs of rare, aberrant rearrangements of Ig V gene segments to TCRγ have been observed in a shark thymocyte cDNA library (M. Criscitiello and M. Flajnik, unpublished data), we conclude that factors capable of binding the Ig promoter (and perhaps eliciting local chromatin remodeling [37]) could be present in thymocytes.

**Figure 9:**
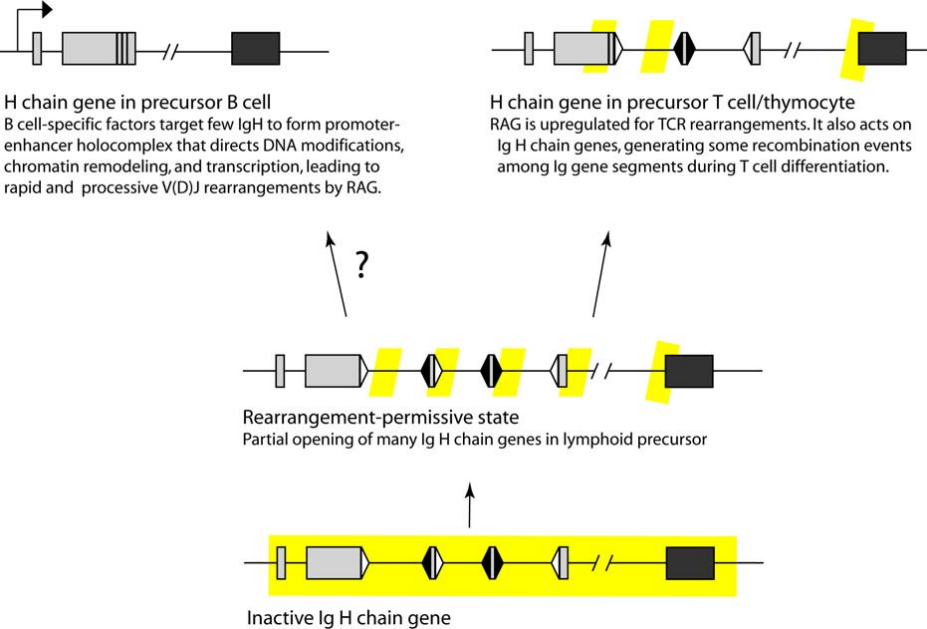
Model of Shark IgH Activation during Lymphocyte Development Shark IgH are in rearrangement-permissive state in precursor lymphocytes but full accessibility to recombinase requires chromatin and DNA modifications mediated by B cell lineage-specific nuclear factors.

Most recently, transcription has been shown correlated with rearrangement competence and induction of chromatin changes [11]. One commentary [38] speculated on the connection between transcription, chromatin remodeling, and recruitment of RAG, pointing out that RAG2 contains a methyl lysine-binding region that may act as a reader for the histone code of the chromatin and thus may act differentially depending upon the pattern of the histone modifications. It is currently thought that the formation of a Ig/TCR promoter-enhancer holocomplex, consisting of a complex of nuclear factors-DNA interaction, directs the chromatin remodeling and DNA modifications that promote chromatin interaction with RAG [39,40]. We propose that the limited number of rearranged IgH per shark B cell is a result of infrequent formation of the holocomplex, which contains lineage-specific factors. These ideas are summarized in Figure 9.

In the absence of B cell-specific factors participating in this holocomplex, IgH in shark precursor lymphocytes may still achieve alternative states of accessibility that are not optimal but not prohibiting for unregulated rearrangement. We propose that a quasi-activated level of chromatin accessibility can exist, supports interaction with RAG, and has distinguishable characteristics.

## Materials and Methods

### Animals.

Shark-JS, -GR, -J, -Y, -BL, and -PI (G. cirratum) were captured off the coast of the Florida Keys and maintained in artificial seawater at approximately 28 °C in large indoor tanks at the National Aquarium at Baltimore. Shark-GR was 7 y of age at the time of bleeding. Whole blood was obtained from the caudal sinus and passed through a Ficoll gradient to separate PBL from RBC. Shark-JS was about 5–6 y of age when sacrificed, and its organs were harvested and frozen. Shark-PI was 3–4 y of age. The thymus was dissociated, passed through a cell strainer mesh (Falcon 2235), and subjected to magnetic cell sorting (see below). DNA and RNA were obtained from PBL and frozen tissues using routine procedures. DNA can be extracted from the RBC, which are nucleated.

### PCR and probes.

There are three Group 2 genes, *G2A–C*, formerly called *V2–4* in [18] (NCBI accession numbers DQ192493, DQ192494, and DQ857389). Mismatches in the primers caused the pseudogene (*G2C*) not to be amplified in this study. Group 2-specific primers used to detect recombined DNA of *G2A* and *G2B* include oligonucleotides targeting the leader (V18–1) or the leader intron (Int, 5′-ATTCAGCAATCAGATAAT-3′). These were paired with primers detecting sequence 3′ of D1 (RSS-D1, 5′-GAATGAGGATGTCGGTAT-3′) or in J_H_ (JH2, 5′- TCACGGTCACCATGGT-3′). Primers for the intersegmental sequence between the two D genes (IntDD-F, 5′-GACGATTCAGAACATAGC-3′) and the unique 5′end of the G2-V2 Cμ2 (V18C2–3′, 5′-CGGAGGGTCACCGTTTCC-3′) detected transcripts from partially rearranged Group 2.

The names of the probes are in lower case (e.g., ns3v probe to the NS3 L chain V region gene, ns4c probe to NS4 L chain C exon). The vh probe (vh: V18–1 5′-ACCAGAATGACGACGATG-3′ and V18–2 5′-GTCTTCGATCTTCAGGC-3′, 461 bp), although derived from a Group 2 sequence, cross-hybridizes with all nurse shark V_H_ [18]. Locus-specific probes can be obtained by using the intersegmental sequences, which are relatively nonconserved among the H chain subfamilies. Probes (Figure 2) for the V-D and D-J intervening DNA (vd2: primers IntVD-F 5′-GTACATTGCACCGTAAAC-3′ and IntVD-R 5′-CGCTCATTCTCTGTTC-3′, 352-bp PCR product; dj2: IntDJ-F 5′-ACAGTGCAGTGTTTACT-3′ and IntDJ-R, 5′-TCACGGTAAATCGTCATC-3′, 239 bp) that were generated from the bacteriophage V18 [22] carrying a *G2A* gene will detect only the three Group 2 loci, *G2A*, *G2B*, and *G2C* (Figure S3). A probe to the conserved cDNA Cμ membrane sequence was also obtained (mem: mem1, 5′-GATTCGATAGATCACACT-3′ and mem2, 5′-AAACAGGACTGATTGTAT- 3′, 216 bp).

Probes to the three nurse shark L chain types NS4 [41], NS3 [26], and NS5 [29] were described previously, and probe names specify whether they detect the V or C sequence (ns3v, ns3c, etc.) ns3v hybridizes to the germline-joined VJ genes of the nonrearranging NS3 L chains [26] and used to standardize DNA on genomic Southern blots because the position of the 3.7-kb band did not overlap with any of the H chain probes. Nurse shark TCRβ C region was cloned from genomic DNA (TCRB-CF, 5′-TCACCAGCAGAGCTGAGA-3′; TCRB-CR, 5′-ATACAGGATGCTCTTGCA-3′). Shark nucleotide diphosphate kinase (ndpk: NDK-F, 5′GGTAACAAGGAACGAACC-3′; NDK-R, 5′-AAAGTTAGTTTATTGTAG-3′) was cloned using PCR primers derived from the available sequence (accession number M63964) [42].

The blots were subjected to autoradiography, and signal intensities of bands were quantified using a Storm 860 phosphorimaging system with ImageQuant software (Molecular Dynamics).

### Separation of membrane-bound IgM-positive cells by magnetic cell sorting.

For IgM+ selection, the buffy coat was resuspended in a mixture of shark IgM-specific mAbs (CB5, CB11, and CB16; [43]), and then with goat-anti-mouse IgG Microbeads (Miltenyi Biotec). Approximately 1.5–5 × 10^7^ cells were collected after two rounds of column purification (Miltenyi Biotec). The negative population was collected as the “flow-through” from the first round of magnetic activated cell sorting (MACS). The positive cells were small and round (lymphocyte-like) cells, whereas the negative population contained cells of different shapes and sizes. Thymocytes were mixed in medium containing a mAb specific for nurse shark NS4 L chain C region (LK14; [44]; E. Hsu, unpublished data), and the L chain-negative cells were collected as “flow-through” from the MACS LS column.

### Single-cell PCR.

Cells were collected after magnetic cell sorting, and RBC were obtained from the same individual for negative controls. Single cells were picked by hand under an inverted microscope with a finely drawn microcapillary pipette (Fisherbrand, #21-164-2G). MAC-sorted lymphocytes were picked, alternating with RBC from another dish; the pipette was rinsed three times in between. The cell was deposited in a 1–1.5-μl volume in shark PBS; 5 μl of lysis solution (1× PCR buffer, 10 mM DTT, 0.5% NP40) was added, topped by mineral oil, and the tubes were heated at 65 °C for one minute to break the nuclear membrane. The tubes were stored at −20 °C until needed. One hundred microliters of 1× PCR solution with dNTP, 0.5 units AmpliTaq (Roche) and primers targeting the V_H_ and J_H_ sequences of Groups 1–5 (two 5′ primers: 20% GR1, 5′-GTTTCTCTACCTCAGCAAT-3′ and 80% GR2–5, 5′-GTTAGTCTMCCTCTGGAAT-3′ with the 3′ primer JH5, 5′-TCACIGTCACCATGGT-3′) were added and the reactions run for 39 cycles at 95 °C 1 min, 58 °C 1 min, 72 °C 1 min, and in the 40th cycle the elongation step was prolonged to 15 min. In the nested reaction, one microliter of the PCR products was added to 50 μl of a second mixture containing two 5′ primers (20% VG1, 5′-AAGGTGTCCAATCGCAA-3′ and 80% VG2–5, 5′-AAGGTGTCCAGTCGGAG 3′) with the 3′ primer JH6 (5′-TCACCATGGTYCCTTGT-3′), and this reaction was run for 20–30 cycles at 95 °C 1 min, 54 °C, 1 min, 72 °C 1 min; again the elongation step was prolonged in the last cycle. The DNA patterns were identical for 20, 25, and 30 cycles. The universal 5′ primers used in the first PCR round are located in the leader intron whereas the nested universal 5′ primers are in FR1 of the V_H_, about 60 bp downstream.

There are two sets of nested Group-specific reactions used to analyze the B cells, one set to identify the 3R fragments observed above, the other to ascertain which IgH remained in GL configuration. For both, the first-round PCR samples were subjected to ExoSAP-IT (USB) to remove remaining “GR” primers. Six microliters of the PCR sample was incubated with 2 μl of ExoSAP-IT for 15 min at 37 °C, followed by inactivation for 15 min at 80 °C. One microliter of the product was used in a 50-μl PCR reaction. For nested reactions to ascertain VDJ identity, the 5′ primers targeted unique, Group-specific sequences in the leader intron, up to 15 bp downstream of the GR primers (G1: Fam1, 5′-AATGTAAAAGACTCAGCC-3′ used at 58 °C; G2: Int, at 58 °C; G3: GR3N2, 5′-TCATGGATTTTTTCATCT-3′ at 54 °C; G4: Fam4, 5′-AATCATTTCATCAGTAAC-3′ at 54 3 °C; G5: Fam5, 5′-GGCTCAGGATTCATTTCG-3′ at 54 °C). In combination with the JH6 primer, 3R products of 400–440 bp were amplified.

The Group-specific primers for GL configuration targeted intersegmental sequences in V-D and D-J. Both 5′ and 3′ primers are specific for the Group, and PCR products of 1.1–1.2 kb were obtained at 58 °C: G1 (G1DF: 5′-CTGTGCAAAAAGCCACG-3′, G1JR: 5′-TGTCCCCAGTGATCAAG-3′; 1,226 bp); G2 (FD2–1: 5′-CACTTTGTACATTGCACC-3′, RD2: 5′-AATAACTGGCTCTGCACG-3′; 1,154 bp); G3 (G3DF: 5′-AACAATGGCTGGACACG-3′; G3JR: 5′-CCCCAGTTACCGAAGTC-3′; 1,242 bp); G4 (G4DF: 5′-ACCACAGAACGAGGAAG-3′; DR3/4: 5′-GCAAAACAAAATCACGAC-3′; 1,143–1,147 bp); G5 (G5DF: 5′-AACAACGGGTGGACCCG-3′; G5JR: 5′-TTGTCCCCAGTAACCGG-3′; 1,224 bp). The cycling parameters for all nested reactions were the same, except for the annealing temperatures.

## Supporting Information

Figure S1 Junctions from Heavy Chain Genomic Rearrangements Junctions from H chain genomic rearrangements. PCR products from shark-JS spleen (JS) and one from thymus (JSTh32) genomic DNA, obtained using the Int/JH2 primers, were cloned into pGEM and sequenced. Top, V sequences containing a single rearrangement event, junctions from VD (from clone containing VD-D-J), DD (V-DD-J), DJ (V-D-DJ) joins are shown. Bottom, V sequences with two rearrangement events, VDD (VDD-J), VD and DJ (VD-DJ), and DDJ (V-DDJ). The reference sequences consist of the flanks of the V_H_ and the J_H_ gene segments and the coding regions of D1 and D2. The two Group 2 genes, *G2A* and *G2B*, differ in the J_H_ flank, as indicated by position R (A or G, respectively). The cloned junctions are aligned with the flanks, and the trimmed positions indicated with dashes, for gaps. Retained portions of D1 and D2 are aligned, and other sequences are assigned as N or P nucleotides. GL is the germline sequence.
(28 KB DOC)

Figure S2 Magnetic Cell Sorting of sIgM+ Nurse Shark PBL Sample assay on nurse shark leukocyte population enriched for sIgM expression (see Materials and Methods). To ascertain enrichment of B cells, the DNA samples were subjected to PCR with primers detecting rearrangement at IgH, IgL, and TCR. DNA samples from the IgM+ (B cell enriched), flow-through (T cell enriched) and erythrocyte (RBC) cell populations were tested for TCRβ rearrangements. PCR reactions were performed with TCRBVF/TCRBJ1, primers in FR2 and J, and dilutions of the DNA (250 ng/reaction, 63 ng, 16 ng, 4 ng, and 1 ng) and run for 40 cycles. The rearranged fragment is on the average about 245 bp (arrow, VDJ). There is some signal of that size in some IgM+ and RBC samples, confirmed by TCRV hybridization, but neither is comparable with the signal obtained in the T cell-enriched population at even the highest dilution. Whether this signal arises from some contaminating T cells or an undefined cell subpopulation or nonfunctional TCR rearrangements in the sIgM+ population is not clear, but its representation is minor. Rearranged H (Int/JH2) and L (NS5LI/NS5JI) chain signals were obtained in the IgM+ and flow-through samples, but not RBC; the flow-through also contains B cells, as might be expected (unpublished data).
(2.47 MB TIF)

Figure S3 Hybridization Patterns of Probes Used in This Study RBC DNA from shark-JS was digested with HincII (H) or with a combination of BamHI/NcoI (B/N), electrophoresed on a 1.2% TBE gel, transferred to HyBond-N filter (Amhersham), and incubated with probes that hybridize to all V_H_ (vh) or specifically to Group 2 (vd2, dj2) or Group 5 (dj5) genes. Hybridizations with the specific probes were done under stringent conditions (72 °C hybridization and washes). The vh, vd2, and dj2 probes were used in experiments shown in Figures 2–4, 8, and S4, the dj5 probe in Figure S7. The dj5 probe was generated from Group 5 GL sequence and the primers DJF-2 (5′-TCAGTGTKTACTTTTAC-3′) and DJR-2 (5′-ATCAMGAYAWAYCTTCA-3′). The first lane (vh probe, HincII digest) is from [23].
(2.6 MB TIF)

Figure S4 Single-Cell Thymocyte PCR, Group 2 Genes (A) The nested single-cell PCR was performed with a 5′ primer specific for Group 2 (Int) and JH6.
(B) The PCR products were analyzed by use of probes to the V_H_ sequence (vh) and the Group 2 intersegmental regions V_H_-D1 (vd2), D1-D2 (dd2), and D2-J (dj2). The PCR products are: Lane 1. RBC, GL (vd2+, dd2+, and dj2+). Lane 2. thymocyte, 1R, VD-D-J (dd2+ and dj+). Lane 3. thymocyte, 2R, VDD-J (dj2+). Lane 4. RBC, GL. Lane 5. thymocyte, GL, V-D-D-J (vd2+, dd2+, and dj2+); 1R, V-DD-J (vd2+ and dj2+); 2R, V-DDJ (vd+). Lane 6. thymocyte, 2R, VDD-J (dj2+). Lane 7. RBC, GL. Lane 8. thymocyte, 2R, VDD-J (dj2+); 3R (vh+). Lane 9. thymocyte, 2R, VDD-J (dj2+). Lane 10. RBC, GL. Lane 11. thymocyte, 1R, V-DD-J (vd2+ and dj2+). Lane 12. thymocyte, 2R, VDD-J (dj2+). Lane 13. vh, vd, dd, dj negative. Lane 14. thymocyte, 2R, VDD-J (dj2+). Lane 15. thymocyte, 2R, VDD-J (dj2+). Lane 16. RBC, GL. Lane 17. thymocyte, GL, V-D-D-J (vh+, vd2+, dd2+, and dj2+). Lane 18. thymocyte, 1R, V-DD-J (vd2+ and dj2+); 2R VDD-J (dj2+).
(2.67 MB TIF)

Figure S5 Single-Cell Thymocyte PCR, Group 1 Genes (A) The nested single-cell PCR was performed with a 5′ primer specific for Group 1 IgH (VG1: 5′-AAGGTGTCCAATCGCAA-3′) and JH6.
(B) The PCR products were characterized by a combination of hybridization with vh probe and a series of restriction enzyme digests. Only Group 1 and Group 5 V_H_ contain a BamHI site; only the Group 1 gene contains PvuII and NcoI sites.
(C) Examples of restriction enzyme analyses of RBC 1 (control) and thymocytes 5, 8, 12, 15, and 18. For example, thy 18 contains ApaLI and PvuII sites, but not NcoI; being the size of 1R, it is thus a V-DD-J configuration. The PCR products are: Lane 1. RBC, GL. Lane 2. vh negative. Lane 3. vh negative. Lane 4. RBC, GL. Lane 5. thymocyte, 2R, VDD-J (BamHI+ and ApaLI+). Lane 6. vh negative. Lane 7. RBC, GL. Lane 8. thymocyte, 1R, V-DD-J (BamHI+, ApaLI+, and PvuII+). Lane 9. thymocyte, 1R, V-DD-J (BamHI+, ApaLI+, and PvuII+); 2R, VDD-J (BamHI+ and ApaLI+). Lane 10. RBC, GL. Lane 11. vh negative. Lane 12. thymocyte, 1R, V-DD-J (BamHI+, ApaLI+, and PvuII+); 2R, VDD-J (BamHI+ and ApaLI+). Lane 13. vh negative. Lane 14. thymocyte, GL. Lane 15. thymocyte, 1R, V-DD-J (BamHI+, ApaLI+, and PvuII+). Lane 16. vh negative. Lane 17. vh negative. Lane 18. thymocyte, 1R, V-DD-J (BamHI+, ApaLI+, and PvuII+).
(5.11 MB TIF)

Figure S6 Single-Cell Thymocyte PCR, Group 4 Genes (A) The nested single-cell PCR was performed with a 5′ primer specific for Group 4 (FAM 4: 5′-AATCATTTCATCAGTAAC-3′) and JH6.
(B) The PCR products were characterized by use of probes to the V_H_ sequence (vh) and restriction enzyme analyses. There are an unknown number of Group 4 genes in shark-PI, but there are at least four, and all of them share these sites.
(C) Examples of restriction enzyme analyses on thymocytes 15, 17, and 8, using EcoRI (left) and SspI and ApaLI (right). Cell 15 contains two 2R species, one being EcoRI positive (V-DDJ) and the other ApaLI positive (VD-DJ). Cell 17 contains only one 2R type that is SspI positive (VDD-J) and a 3R. Cell 8 contains three 2R species, one is EcoRI positive (V-DDJ), one is ApaLI+ (VD-DJ) and the other SspI positive (VDD-J). The PCR products are: Lane 1. RBC, GL. Lane 2. thymocyte, 2R, VDD-J (SspI+). Lane 3. thymocyte, 2R, VD-DJ (ApaLI+) and VDD-J (SspI+). Lane 4. RBC, GL. Lane 5. thymocyte, 2R, VD-DJ (ApaLI+) and V-DDJ (EcoRI+). Lane 6. thymocyte, 2R, V-DDJ (EcoRI+). Lane 7. vh negative. Lane 8. thymocyte, 2R, V-DDJ (EcoRI+), VD-DJ (ApaLI+) and VDD-J (SspI+). Lane 9. thymocyte, (cloned; see Table 2). Lane 10. RBC, GL. Lane 11. thymocyte, 1R, VD-D-J (ApaLI+ and SspI+) and V-D-DJ (EcoRI+ and ApaLI+); 2R, VD-DJ (ApaLI+). Lane 12. vh negative. Lane 13. vh negative. Lane 14. thymocyte, 1R, VD-D-J (ApaLI+ and SspI+) and V-D-DJ (EcoRI+ and ApaLI+); 2R, VDD-J (SspI+); 3R. Lane 15. thymocyte, 2R, VDD-J (SspI+) and V-DDJ (EcoRI+). Lane 16. RBC, GL. Lane 17. thymocyte, 2R, VD-DJ (ApaLI+); 3R. Lane 18. thymocyte, 3R.
(4.30 MB TIF)

Figure S7 Single-Cell Thymocyte PCR, Group 5 Genes (A) The nested single-cell PCR was performed with a 5′ primer specific for Group 5 (FAM 5: 5′-GGCTCAGGATTCATTTCG-3′) and JH6.
(B) The PCR products were characterized by use of probes to the Group 5 D-J region (dj5) and restriction enzyme analyses.
(C) Restriction enzyme digests of PCR products from RBC control and thymocytes. Top, Sca I-digested samples; bottom, EcoRV. The PCR products are: Lane 1. RBC, GL (dj5 probe positive, BamHI+, ScaI+, and EcoRV+). Lane 2. thymocyte, 2R, V-DDJ (dj5 probe negative, BamHI+, and ScaI+). Lane 3. thymocyte, 2R, V-DDJ (BamHI+ and ScaI+). Lane 4. RBC, GL. Lane 5. thymocyte, 2R, V-DDJ (BamHI+ and ScaI+). Lane 6. dj5 negative. Lane 7. RBC, GL. Lane 8. thymocyte, 3R (BamHI+). Lane 9. thymocyte, 3R. Lane 10. RBC, GL. Lane 11. thymocyte, 2R, V-DDJ (BamHI+ and ScaI+). Lane 12. thymocyte, 3R. Lane 13. dj5 negative. Lane 14. thymocyte, 3R. Lane 15. thymocyte, 3R. Lane 16. dj5 negative. Lane 17. thymocyte, 3R. Lane 18. thymocyte, 2R, V-DDJ (BamHI+ and ScaI+).
(5.53 MB TIF)

Figure S8 End-Labeled Single-Cell VDJ Characterized by CDR3 Length Heterogeneity DNA samples from single-cell PCR of B cells were digested with NcoI, the recognition site being present in the JH6 primer that is used in the nested round of PCR (3R about 350–370 bp; see Figure 4, bottom). NcoI leaves a recessed 3′ end that can be filled in with ^32^P-CTP. The end-labeled samples were denatured and loaded on a sequencing gel along with the sequence of M13mp18 (lanes marked GATC). The blue dot at the bottom marks the C position at 339 bases of the phage, sequenced with the −40 primer (Sequenase Version 2.0, USB). There is some labeling in the absence of NcoI digestion (first and last lanes, L7 and K5). Lanes L7, L17, K8, and K5 show one band, whereas K11 show two (cloned as VDJ from Groups 4 and 5). Lane N11 was an artifact at 450 bp (determined by sequencing). The technique had been devised as a method of distinguishing Ig sequences utilizing the variability at CDR3 [45].
(1.54 MB TIF)

Figure S9 Testing the Number of VDJ Species per 3R Band Every non-GL band found in the single-cell reactions (Figures 5–7) was cloned into pGEM vector after excising the band from an agarose gel and eluting the DNA using Qiagen columns. Usually the same sequence was obtained repeatedly in three to five clones. We determined whether there was one or more VDJ in the 3R band the following way. (1) Single bacterial colonies from the transformation would be suspended in LB medium and an aliquot subjected to PCR using the T7 and Sp6 primers to amplify the inserts. The 15–40 such PCR products would be digested with restriction enzymes, using a site in CDR3 that was found in the original sequence (listed in Figure S11). Every nondigested band was directly sequenced. (2) The original PCR product (both the universal primer product as in Figure 5 and the Group-specific product in Figures 6 and 7) would digested with the same restriction enzyme to ascertain whether all components of the band were digested. This is illustrated in the Panel KM3/G2 where the PCR product raised by Group 2-specific 5′ primer Int (and JH6) was completely digested with Mwo I. In the panel KM5/G4 the first sequenced plasmids contained a VDJ with an EcoRV site. The 3R fragment did not digest completely (as shown in lane 2), which suggested that a second, EcoRV-negative VDJ was present. Among 45 bacterial colonies, 18 carried an EcoRV site and 27 did not. The latter clones were grown up and sequenced, showing VDJ that carried a HaeIII site. Thus the cell sample KM5 carried two rearrangements of the *G4* subfamily. Sometimes a GL sequence was amplified along with the 3R band as in panel KM15/G5. The VDJ carried two MseI sites, one in the V_H_ and the second in CDR3, as diagrammed below the photograph of the gel. The 441-bp VDJ is expected to be digested by MseI into three fragments, 264 bp, 110 bp, and 67 bp. The 110-bp fragment is diagnostic and indicated with an arrow. There are multiple sites in the GL fragment, but these are present at a fraction of the VDJ and do not interfere with the interpretation. In summary, all the VDJ listed in Figure S11, except in cell KM5, were the only species present in the 3R band, and the results appeared as shown for sample KM3 or KM15. The enzymes used are listed in Figure S11.
(2.35 MB TIF)

Figure S10 Unrearranged IgH Genes in Single B Cells First-round PCR products from single B cells were subjected to nested PCR with primers in V-D and D-J that would amplify unrearranged sequence, bracketed in top diagram. The Group-specific primers separately amplified: Group 1 (1,226 bp), Group 2 (G2A, G2B: 1,154 bp), Group 3 (1,242 bp), Group 4 (G4A, G4D, G4E, G4C/G: 1,144–1,147 bp), and Group 5 (1,224 bp). I. Group 1 GL contains unique PvuII site in the V-D region, which can be detected as shown in agarose gel at right. GL sequences were amplified from single cells, which were confirmed to be *G1* by presence of PvuII site. C is control, no enzyme. II. Group 2A GL sequence can be distinguished from Group 2B by two sites, NdeI and EcoRI, which are absent in *G2B*. In the gels, sample 1 carries *G2B* but not *G2A*; and sample 2 carries *G2A* but not *G2B*; the rest (3–5) carry both *G2A* and *G2B*. III. Group 3 GL contains an ApaLI site in the D-D region (as shown) but is negative for PvuII, EcoRI, and EcoRV (unpublished). IV. The four Group 4 GL sequences can be distinguished by a series of restriction enzyme digestions. A combination of SspI and EcoRI demonstrates whether *G4A* (607, 273, and 265 bp) are components of the PCR products. The bolded fragment, 607 bp, is indicated by the blue bar in panel IV, *G4A*, and is present only (arrows) in samples 1 and 4 in the first gel at left. In this photograph, the marker lane was transposed closer to the four sample lanes than it was in the original gel. To distinguish whether the *G4* samples whose PCR products contain *G4D* or *G4A* or both, they were incubated with SalI (G4D: 796, 348 bp, G4A: 1019, 126 bp). The diagnostic band in the SalI gel are marked with arrows. C is control, no enzyme. The presence of *G4CG* and *G4E* involve somewhat more complex digestion patterns but the diagnostic bands are distinct. Digestions of the GL PCR product with ScaI and EcoRI provide only *G4CG* with a 186-bp fragment (G4CG: 874, 186, and 87 bp; G4A: 872 and 273 bp; G4D: 1,057 and 87 bp; and G4E: 874 and 273 bp). Samples 1 and 2 but not samples 3 and 4, carry the *G4CG* genes. Digestions of the GL PCR product with HincII and EcoRI provide only *G4E* with a 200-bp fragment (G4E: 856, 200, 73, and 18 bp; G4A: 854, 144, 126, and 18 bp; G4D: 505, 348, and 291 bp; G4CG: 856, 273, and 18 bp). These diagnostic bands are marked with arrows; samples 1 and 2, but not sample 3, carry *G4E*. V. Group 5 GL sequences could be confirmed with the presence of the unique EcoRV site in the D-D region, as shown by gel at the right.
(3.33 MB TIF)

Figure S11 Single-B Cell VDJ Junctions The CDR3 of rearranged VDJ from single B cells shown in Table 3 are aligned under the GL flanks of the V_H_ and the J_H_ gene segments and the coding regions of D1 and D2. The trimmed positions are shown with dashes, for gaps, and other sequences are assigned as N or P nucleotides. The GL sequences were cloned from shark-GR, so that mutated positions could be identified clearly (indicated as lower case). “+” indicates in-frame potentially functional sequence, “non” is in-frame CDR3 with stops (underlined), null symbol indicates out-of-frame sequence. The VDD-J in KS23 has undergone only two rearrangement events. The CDR3-based restriction enzymes listed at the right were used to detect the number of VDJ species per 3R band.
(36 KB DOC)

Figure S12 Transcripts from Partially Rearranged Heavy Chain Genes Show Evidence of Hypermutation Top, diagram showing positions of two of the PCR primer pairs used and the transcripts detected relative to the GL genes. RT-PCR was performed on shark-JS spleen RNA with various PCR primer combinations described in Materials and Methods, and the products were cloned and sequenced. Bottom, representative mutated sequences are aligned to the GL gene G2-V2, whose V_H_, D1, D2, and J_H_ gene segments are labeled. The CDR1 and CDR2 in the V_H_ are underlined; RSS are bolded, as is the leader intron acceptor splice site. Clone A36: Int/JH2 primer pair, two-rearrangement VDD-J sequence (accession number: DQ857392). Clone LVD1–3: Int/RSSD1 primer pair, VD1 sequence, derived from VD-DJ or VD-D-J transcript. Clone DDC4: IntDD-F/V18C2–3′ primer pair, DJC sequence (accession number: DQ857391), derived from transcript carrying VD-DJ or V-D-DJ; its Group 2 C region sequence is not shown. These clones were chosen for the presence of mutations throughout the sequence; no portion was shared with any non-Group 2 sequences. The tandem substitutions are typical of hypermutated shark Ig [26]. Dots denote identity with the reference sequence, dashes gaps. Substitutions are shown in capital letters, N region in lower case in front of the D or J_H_ sequence. Insertions are marked with arrow. Sites of the PCR primers are indicated in brackets.
(856 KB TIF)

Figure S13 Genomic Rearrangements of NS5 L Chain Diagram at top: organization of the NS5–2 L chain locus and location of the primers. Boxes represent coding sequences of leader, V and J gene segments and C exon, filled triangle an RSS with 12-bp spacer, open triangle an RSS with 23-bp spacer. Bottom: 1.5% agarose gel, PCR was performed using primers in the leader intron and 3′ of JL, that detect the NS5–2 L chain gene [29], GL V-J (arrow, GL at 880 bp) and rearranged VJ (∼490 bp), in spleen DNA (lane 8). The RBC lane was inserted from another position (11th lane) on the same gel.
(1.19 MB TIF)

Text S1 Glossary of Terms (82 KB DOC)

## Abbreviations

GL: germline
H: heavy
Ig: immunoglobulin
L: light
PBL: peripheral blood leukocyte
RBC: red blood cell
sIgM+: surface IgM-expressing
