# Supplementary material for: Immunoglobulin Heavy Chain Exclusion in the Shark
Source: PLoS Biol. 2008 Jun 24;6(6):e157. doi: 10.1371/journal.pbio.0060157 (PMC2435157; doi:10.1371/journal.pbio.0060157)
Supplement: Figure S1 — Junctions from H chain genomic rearrangements. PCR products from shark-JS spleen (JS) and one from thymus (JSTh32) genomic DNA, obtained using the Int/JH2 primers, were cloned into pGEM and sequenced. Top, V sequences containing a single rearrangement event, junctions from VD (from clone containing VD-D-J), DD (V-DD-J), DJ (V-D-DJ) joins are shown. Bottom, V sequences with two rearrangement events, VDD (VDD-J), VD and DJ (VD-DJ), and DDJ (V-DDJ). The reference sequences consist of the flanks of the VH and the JH gene segments and the coding regions of D1 and D2. The two Group 2 genes, G2A and G2B, differ in the JH flank, as indicated by position R (A or G, respectively). The cloned junctions are aligned with the flanks, and the trimmed positions indicated with dashes, for gaps. Retained portions of D1 and D2 are aligned, and other sequences are assigned as N or P nucleotides. GL is the germline sequence. (28 KB DOC) [file pbio.0060157.sg001.doc]

**VH** N/P **D1** N/P **D2** N/P **J** gene segment

# TGTGCAAGAGAC ATACTACAGTGGGT ACATACTGGGATAG ACTATTTTGRTTACTGG

###### VD-D-J

JS13 TGTGCAAGA--- AGTGACTACGTTAT AGTGGGT GL GL

JS31 TGTGCAAGAG-- CCTCC CAGTGGGT GL GL

JS40 ------------ ACCACTTCGG GTGGGT GL GL

JS44 TGTGCAAGAGA- GGGATATGTA TACAGTGGGT GL GL

JS53 TGTGCAAG---- GAT CTACAGTGGGT GL GL

JS74 TGTGCAAGAGA- TC CAGTGGGT GL GL

JS22DD TGTGC------- GAGTTG TACAGTGGGT GL GL

JS24DD TGTGCAAGAGA- CGTGG ACAGTGGGT GL GL

JS26DD TGTGCAAGA--- AC ACTACAGTGGGT GL GL

###### V-DD-J

JS66 GL ATACTACAGT CGGGATTGG TGGGATAG GL

JS6VD GL ATACTACAGTGGGT AGGACT ATACTGGGATAG GL

###### V-D-DJ

JS23DD GL GL ACATACTG ACCC ----TTTTGATTACTGG

**VH**  N/P **D1** N/P **D2** N/P **J** gene segment

# TGTGCAAGAGAC ATACTACAGTGGGT ACATACTGGGATAG ACTATTTTGRTTACTGG

###### VDD-J

JS1 TGTGCAAGA--- ATCA TGGG CCACG ACTGGGATAG GL

JS2 TGTGCAA----- TTAAGCAAT AGTGGG TACTGGGATAG GL

JS18 TGTGCAAGA--- TT GGGT ACA GGGATAG GL

JS22 TGTGCAAGA--- ACG ACTACAGTGGGT ACAGT ACATACTGGGATAG GL

JS25 TGTGCAA----- TTCGATC GTGGGT TC ACTGGGATAG GL

JS27 TGTGCAAG---- T GTGGGT AC GGGATAG GL

JS29 TGTGCAAG---- TACA CTTC TACTGGGATAG GL

JS32 TGTGCAA----- AGGGCCCT CAGTGGGT ACAAGGTTGT GGATAG GL

JS36 TGTGCAAGA--- TTGA ATA TGA TGGGATAG GL

JS43 TGTGCAA----- CCATATAA ATAC TTCTCCCTA TGGGATAG GL

JS45 TGTGCAAG---- GTAGGG ATACTACAGTGGGT ACTTGGG CATACTGGGATAG GL

JS46 TGTGCAAG---- TAT GGT CGGAC ACTGGGATAG GL

JS47 TGTGCAAGA--- T TA TT ACATACTGGGATAG GL

JS48 TGTGCAAG---- CCGAT ATACTACAGTG TCACCC ACTGGGATAG GL

JS49 TGTGCAAGA--- TCG ACTA TGGGATAG GL

JS50 TGTGCAAGAG-- TTGGA TACAG TGGGATAG GL

JS52 TGTGCAAGA--- AA ACAGT T TACTGGGATAG GL

JS54 TGTGCAAG---- GATAA ACAGTGGG GG ACTGGGATAG GL

JS57 TGTGCAAGA--- GGTCGGG ACTACAGTGGGT CCCCATAT CATACTGGGATAG GL

JS65 TGTGCAAGA--- T AGT CAACCTC ACTGGGATAG GL

JS69 TGTGCA------ G CAGT T GGATAG GL

JS70 TGTGCAAGAGAC AG AT CATACTGGGATAG GL

JS73 TGTGCAAGA--- CGT AGTGGGT ACGGGACTTA TGGGATAG GL

JS75 TGTGCAAGA--- AGAAGG CTACAGTG ATC TAG GL

JS76 TGTGC------- TC CTACAGT AG ACTGGGATAG GL

JS79 TGTGCAAGA--- ATACT ACATACTGGGATAG GL

JS80 TGTGCAAGA--- ATCGATATAGGGAT ATACTACAGTG CCATTGGG ACATACTGGGATAG GL

JS82 TGTGCAA----- AGGG GTGGGT CTA GGGATAG GL

JS84 TGTGCAAGAGA- AG AGTGGGT GAA TGGGATAG GL

JS85 TGTGCAAGAG-- GTCGGC ACTACAGTGGGT ACG ACTGGGATAG GL

JS11DD TGTGCAAG---- TACTAC GACCATTTT ATAG GL

JS1VD TGTGCAAGAGAC CT C GGATAG GL

###### VD-DJ

JS8 TGTGCAAGAGAC GGGGAT GTGGGT ACATACTGGGATAG GGG -----TTTGGTTACTGG

JS51 TGTGCAAG---- TCATATCC CAGTGGGT ACATAC GGGACCCA ------TTGATTACTGG

###### V-DDJ

JSTh32 GL ATACTACAGTGG AGGTCTAGGGCATATGT ACATACTGGGATA CCTCCGCCG -----TTTGATTACTGG
