# Supplementary figures and images for: Immunoglobulin Heavy Chain Exclusion in the Shark
Source: PLoS Biol. 2008 Jun 24;6(6):e157. doi: 10.1371/journal.pbio.0060157 (PMC2435157; doi:10.1371/journal.pbio.0060157)

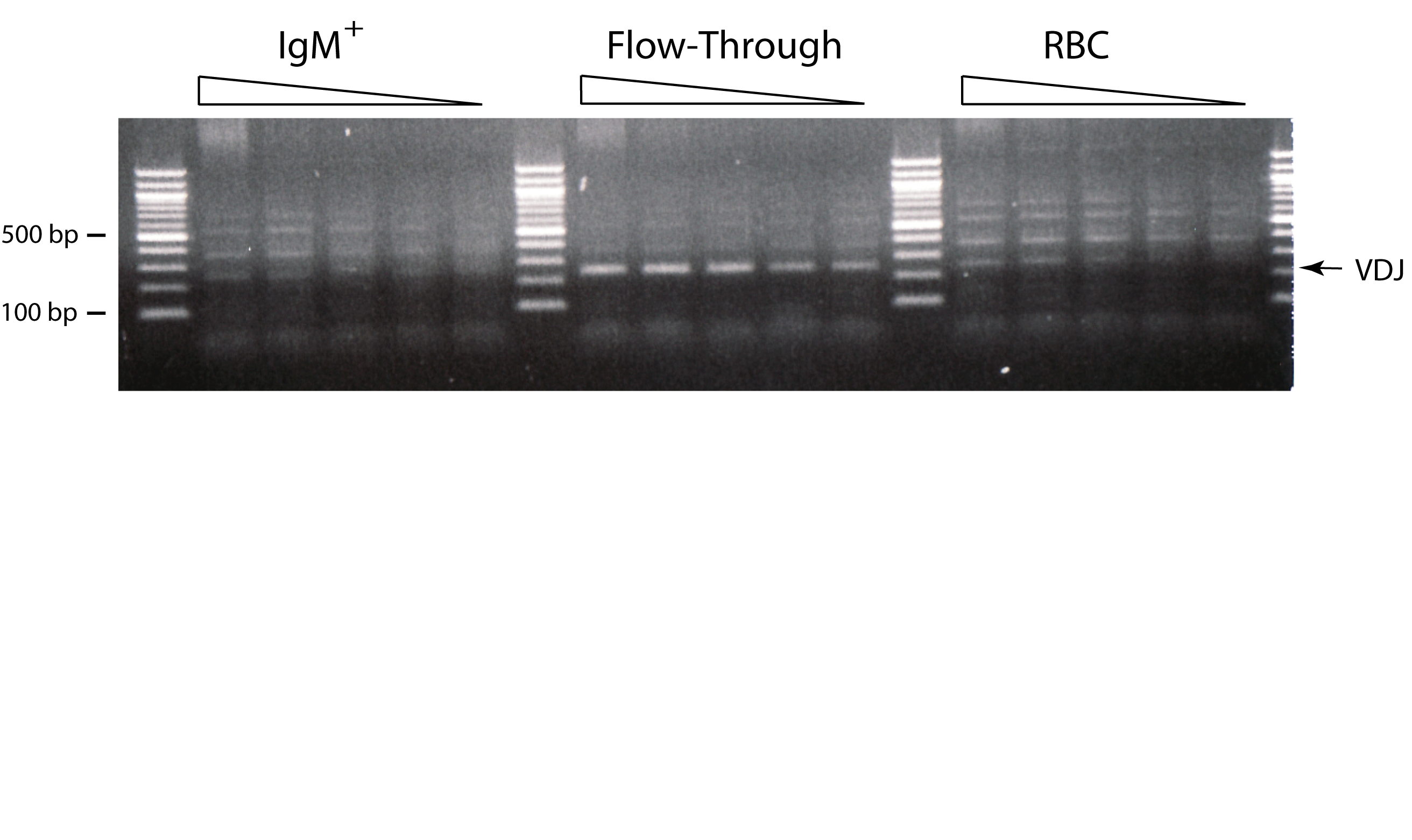

Supplement: Figure S2 — Sample assay on nurse shark leukocyte population enriched for sIgM expression (see Materials and Methods). To ascertain enrichment of B cells, the DNA samples were subjected to PCR with primers detecting rearrangement at IgH, IgL, and TCR. DNA samples from the IgM+ (B cell enriched), flow-through (T cell enriched) and erythrocyte (RBC) cell populations were tested for TCRβ rearrangements. PCR reactions were performed with TCRBVF/TCRBJ1, primers in FR2 and J, and dilutions of the DNA (250 ng/reaction, 63 ng, 16 ng, 4 ng, and 1 ng) and run for 40 cycles. The rearranged fragment is on the average about 245 bp (arrow, VDJ). There is some signal of that size in some IgM+ and RBC samples, confirmed by TCRV hybridization, but neither is comparable with the signal obtained in the T cell-enriched population at even the highest dilution. Whether this signal arises from some contaminating T cells or an undefined cell subpopulation or nonfunctional TCR rearrangements in the sIgM+ population is not clear, but its representation is minor. Rearranged H (Int/JH2) and L (NS5LI/NS5JI) chain signals were obtained in the IgM+ and flow-through samples, but not RBC; the flow-through also contains B cells, as might be expected (unpublished data). (2.47 MB TIF) [file pbio.0060157.sg002.tif]

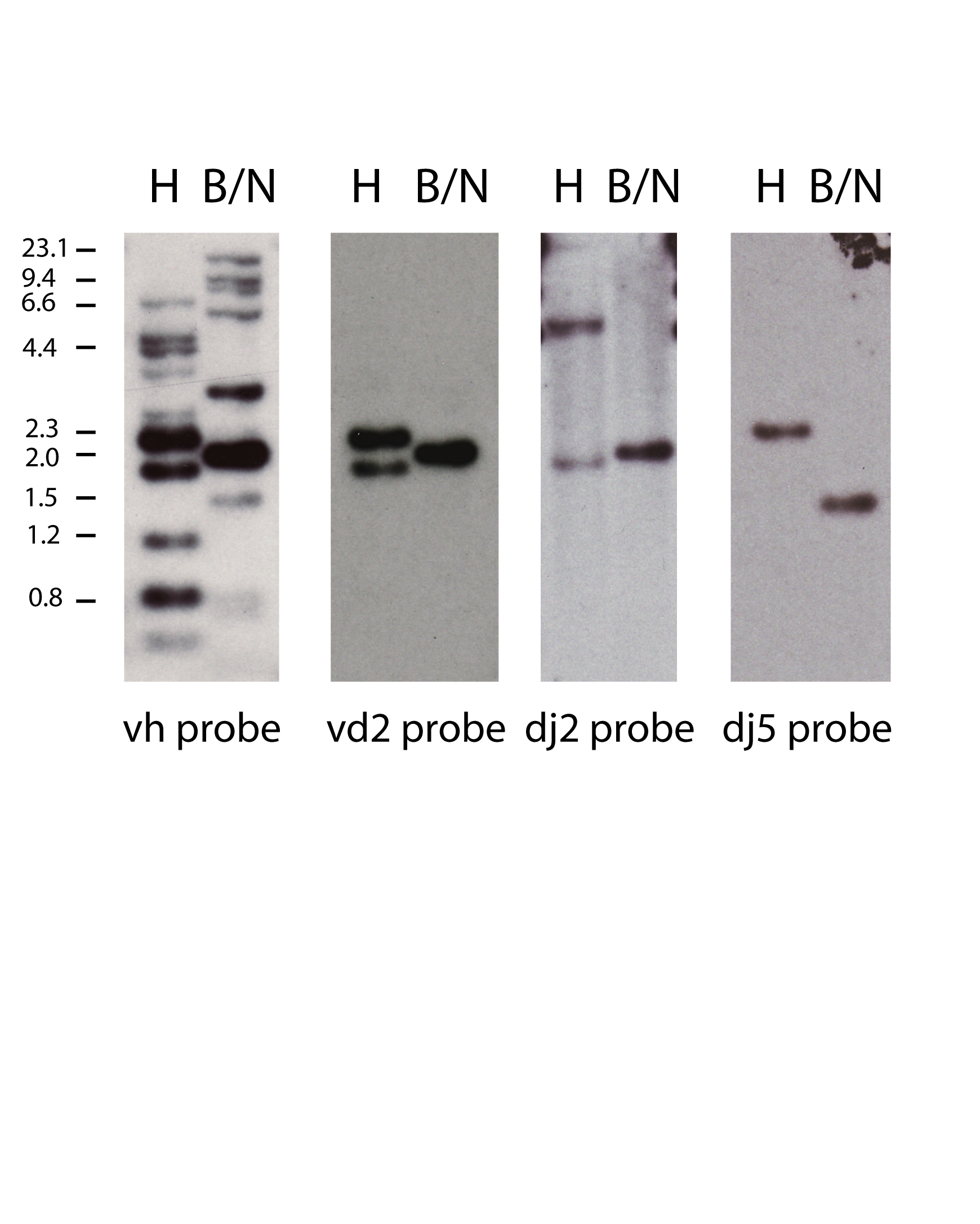

Supplement: Figure S3 — RBC DNA from shark-JS was digested with HincII (H) or with a combination of BamHI/NcoI (B/N), electrophoresed on a 1.2% TBE gel, transferred to HyBond-N filter (Amhersham), and incubated with probes that hybridize to all VH (vh) or specifically to Group 2 (vd2, dj2) or Group 5 (dj5) genes. Hybridizations with the specific probes were done under stringent conditions (72 °C hybridization and washes). The vh, vd2, and dj2 probes were used in experiments shown in Figures 2–4, 8, and S4, the dj5 probe in Figure S7. The dj5 probe was generated from Group 5 GL sequence and the primers DJF-2 (5′-TCAGTGTKTACTTTTAC-3′) and DJR-2 (5′-ATCAMGAYAWAYCTTCA-3′). The first lane (vh probe, HincII digest) is from [23]. (2.6 MB TIF) [file pbio.0060157.sg003.tif]

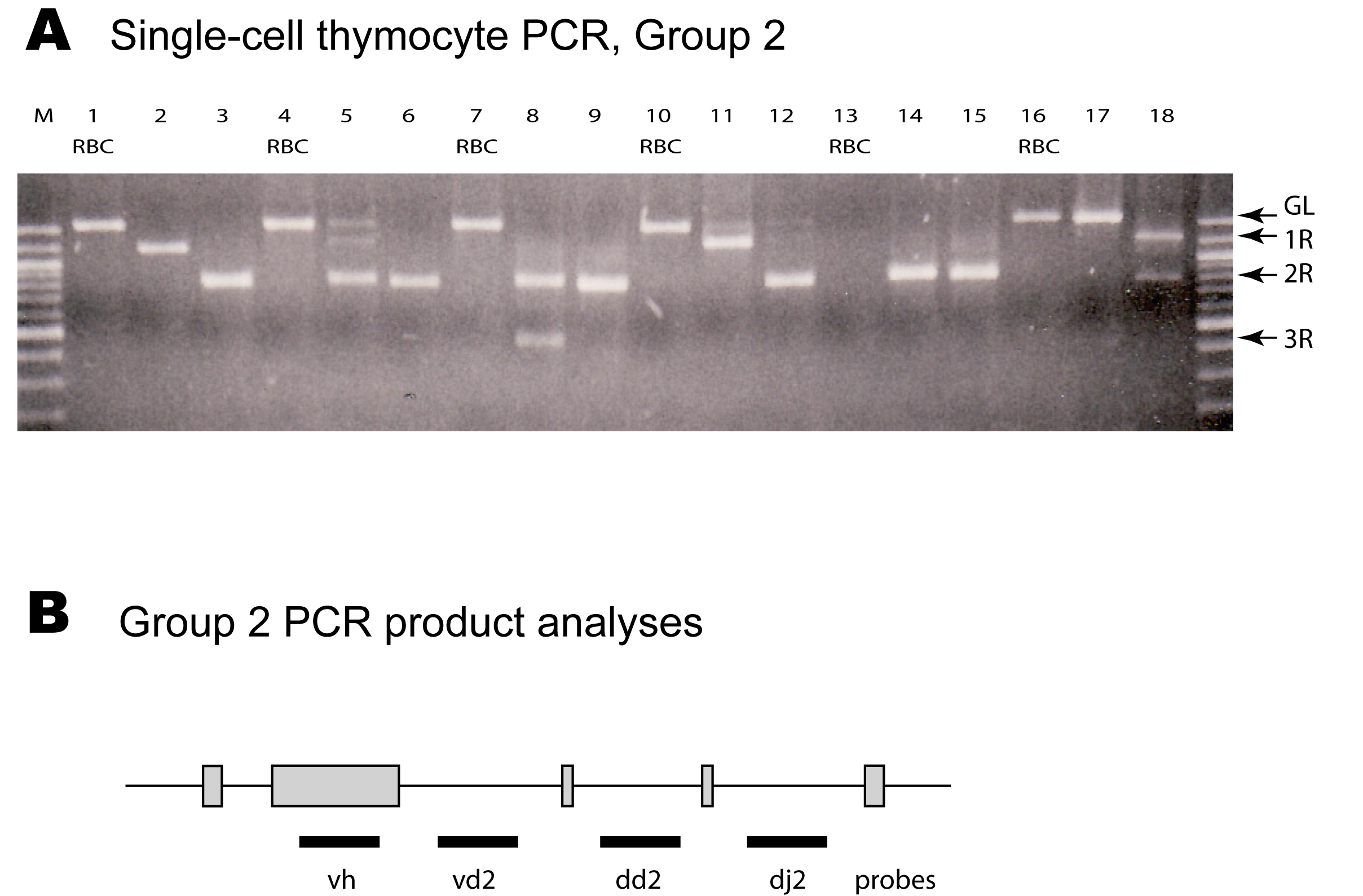

Supplement: Figure S4 — (A) The nested single-cell PCR was performed with a 5′ primer specific for Group 2 (Int) and JH6. (B) The PCR products were analyzed by use of probes to the VH sequence (vh) and the Group 2 intersegmental regions VH-D1 (vd2), D1-D2 (dd2), and D2-J (dj2). The PCR products are: Lane 1. RBC, GL (vd2+, dd2+, and dj2+). Lane 2. thymocyte, 1R, VD-D-J (dd2+ and dj+). Lane 3. thymocyte, 2R, VDD-J (dj2+). Lane 4. RBC, GL. Lane 5. thymocyte, GL, V-D-D-J (vd2+, dd2+, and dj2+); 1R, V-DD-J (vd2+ and dj2+); 2R, V-DDJ (vd+). Lane 6. thymocyte, 2R, VDD-J (dj2+). Lane 7. RBC, GL. Lane 8. thymocyte, 2R, VDD-J (dj2+); 3R (vh+). Lane 9. thymocyte, 2R, VDD-J (dj2+). Lane 10. RBC, GL. Lane 11. thymocyte, 1R, V-DD-J (vd2+ and dj2+). Lane 12. thymocyte, 2R, VDD-J (dj2+). Lane 13. vh, vd, dd, dj negative. Lane 14. thymocyte, 2R, VDD-J (dj2+). Lane 15. thymocyte, 2R, VDD-J (dj2+). Lane 16. RBC, GL. Lane 17. thymocyte, GL, V-D-D-J (vh+, vd2+, dd2+, and dj2+). Lane 18. thymocyte, 1R, V-DD-J (vd2+ and dj2+); 2R VDD-J (dj2+). (2.67 MB TIF) [file pbio.0060157.sg004.tif]

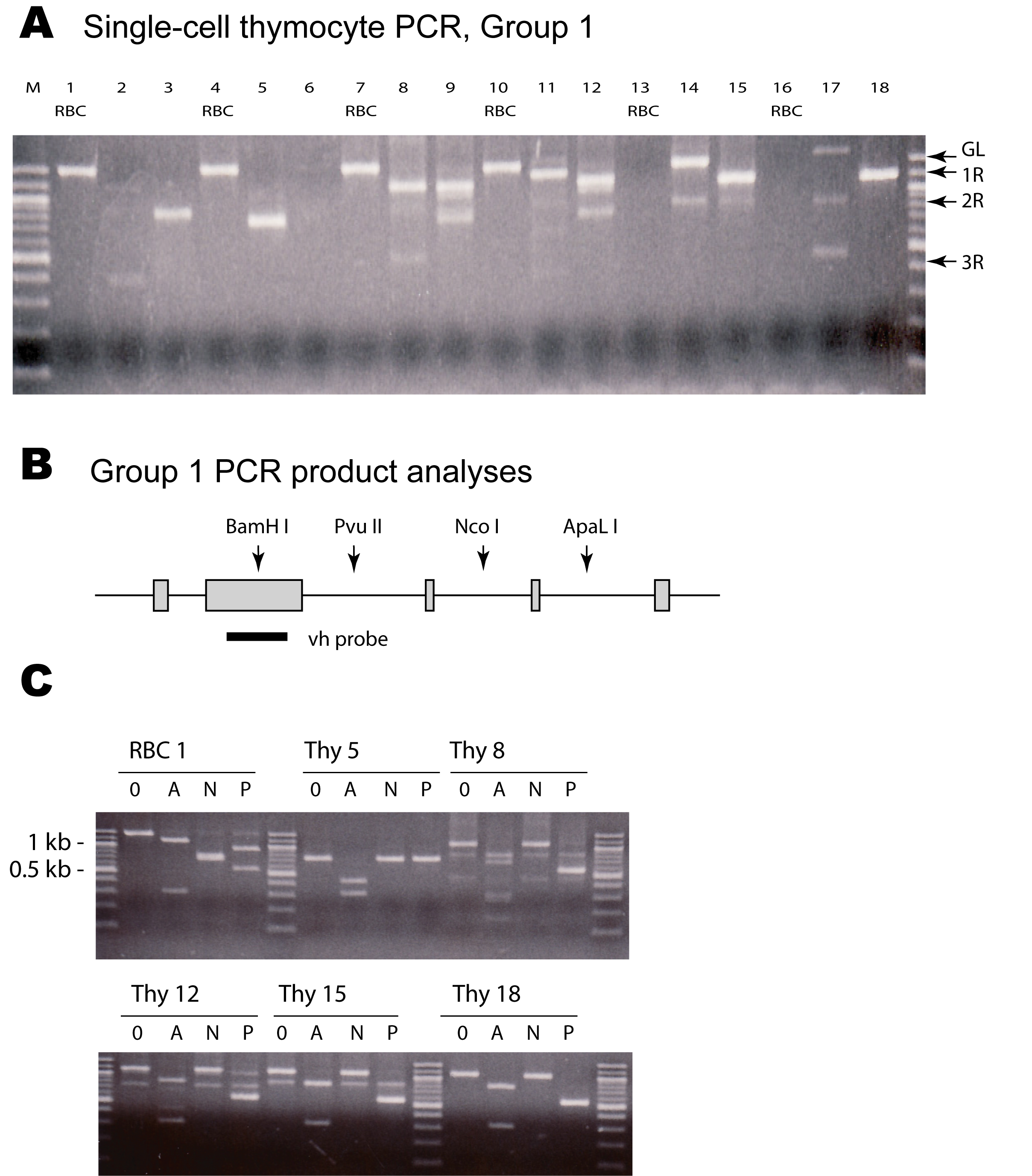

Supplement: Figure S5 — (A) The nested single-cell PCR was performed with a 5′ primer specific for Group 1 IgH (VG1: 5′-AAGGTGTCCAATCGCAA-3′) and JH6. (B) The PCR products were characterized by a combination of hybridization with vh probe and a series of restriction enzyme digests. Only Group 1 and Group 5 VH contain a BamHI site; only the Group 1 gene contains PvuII and NcoI sites. (C) Examples of restriction enzyme analyses of RBC 1 (control) and thymocytes 5, 8, 12, 15, and 18. For example, thy 18 contains ApaLI and PvuII sites, but not NcoI; being the size of 1R, it is thus a V-DD-J configuration. The PCR products are: Lane 1. RBC, GL. Lane 2. vh negative. Lane 3. vh negative. Lane 4. RBC, GL. Lane 5. thymocyte, 2R, VDD-J (BamHI+ and ApaLI+). Lane 6. vh negative. Lane 7. RBC, GL. Lane 8. thymocyte, 1R, V-DD-J (BamHI+, ApaLI+, and PvuII+). Lane 9. thymocyte, 1R, V-DD-J (BamHI+, ApaLI+, and PvuII+); 2R, VDD-J (BamHI+ and ApaLI+). Lane 10. RBC, GL. Lane 11. vh negative. Lane 12. thymocyte, 1R, V-DD-J (BamHI+, ApaLI+, and PvuII+); 2R, VDD-J (BamHI+ and ApaLI+). Lane 13. vh negative. Lane 14. thymocyte, GL. Lane 15. thymocyte, 1R, V-DD-J (BamHI+, ApaLI+, and PvuII+). Lane 16. vh negative. Lane 17. vh negative. Lane 18. thymocyte, 1R, V-DD-J (BamHI+, ApaLI+, and PvuII+). (5.11 MB TIF) [file pbio.0060157.sg005.tif]

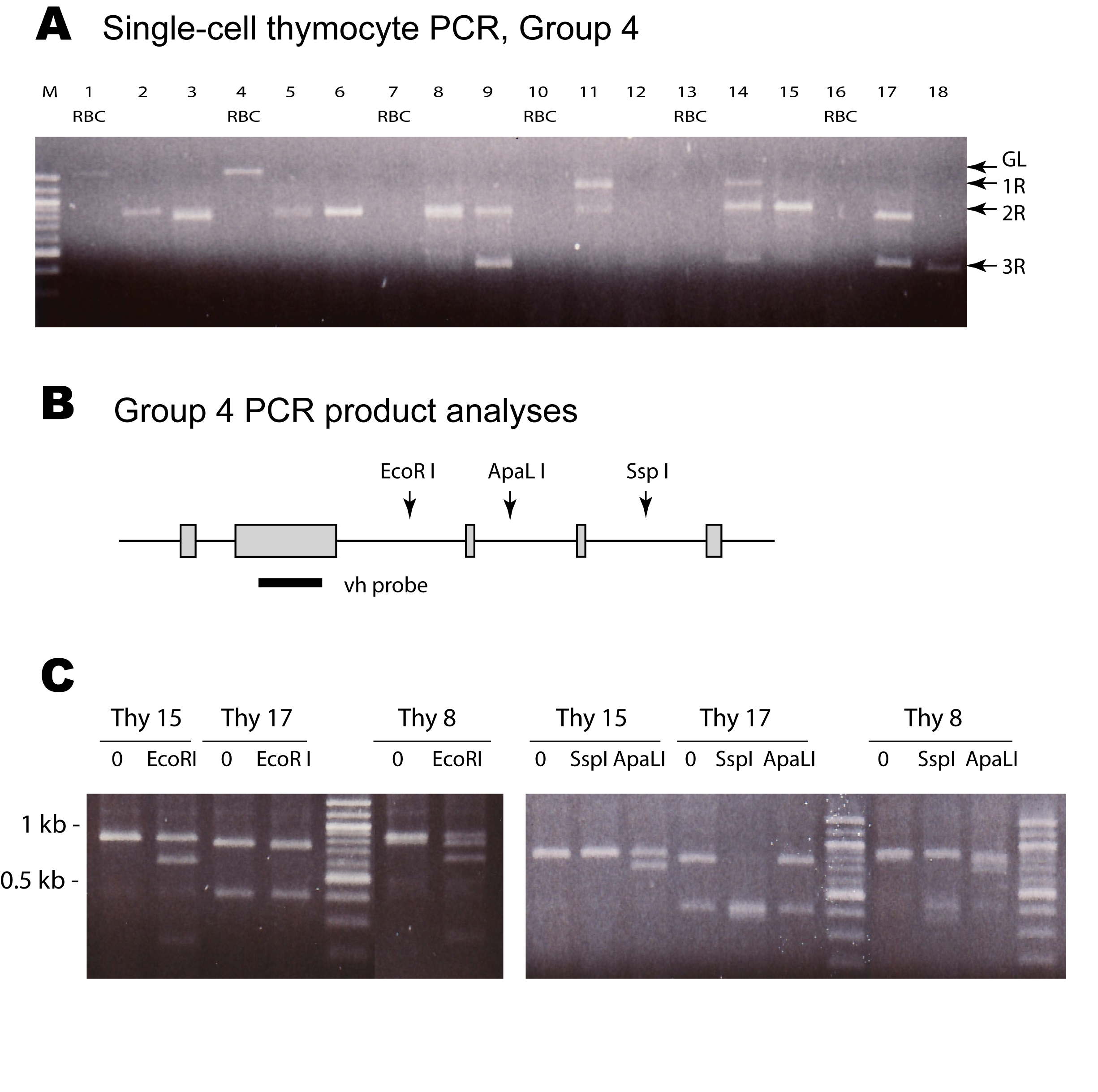

Supplement: Figure S6 — (A) The nested single-cell PCR was performed with a 5′ primer specific for Group 4 (FAM 4: 5′-AATCATTTCATCAGTAAC-3′) and JH6. (B) The PCR products were characterized by use of probes to the VH sequence (vh) and restriction enzyme analyses. There are an unknown number of Group 4 genes in shark-PI, but there are at least four, and all of them share these sites. (C) Examples of restriction enzyme analyses on thymocytes 15, 17, and 8, using EcoRI (left) and SspI and ApaLI (right). Cell 15 contains two 2R species, one being EcoRI positive (V-DDJ) and the other ApaLI positive (VD-DJ). Cell 17 contains only one 2R type that is SspI positive (VDD-J) and a 3R. Cell 8 contains three 2R species, one is EcoRI positive (V-DDJ), one is ApaLI+ (VD-DJ) and the other SspI positive (VDD-J). The PCR products are: Lane 1. RBC, GL. Lane 2. thymocyte, 2R, VDD-J (SspI+). Lane 3. thymocyte, 2R, VD-DJ (ApaLI+) and VDD-J (SspI+). Lane 4. RBC, GL. Lane 5. thymocyte, 2R, VD-DJ (ApaLI+) and V-DDJ (EcoRI+). Lane 6. thymocyte, 2R, V-DDJ (EcoRI+). Lane 7. vh negative. Lane 8. thymocyte, 2R, V-DDJ (EcoRI+), VD-DJ (ApaLI+) and VDD-J (SspI+). Lane 9. thymocyte, (cloned; see Table 2). Lane 10. RBC, GL. Lane 11. thymocyte, 1R, VD-D-J (ApaLI+ and SspI+) and V-D-DJ (EcoRI+ and ApaLI+); 2R, VD-DJ (ApaLI+). Lane 12. vh negative. Lane 13. vh negative. Lane 14. thymocyte, 1R, VD-D-J (ApaLI+ and SspI+) and V-D-DJ (EcoRI+ and ApaLI+); 2R, VDD-J (SspI+); 3R. Lane 15. thymocyte, 2R, VDD-J (SspI+) and V-DDJ (EcoRI+). Lane 16. RBC, GL. Lane 17. thymocyte, 2R, VD-DJ (ApaLI+); 3R. Lane 18. thymocyte, 3R. (4.30 MB TIF) [file pbio.0060157.sg006.tif]

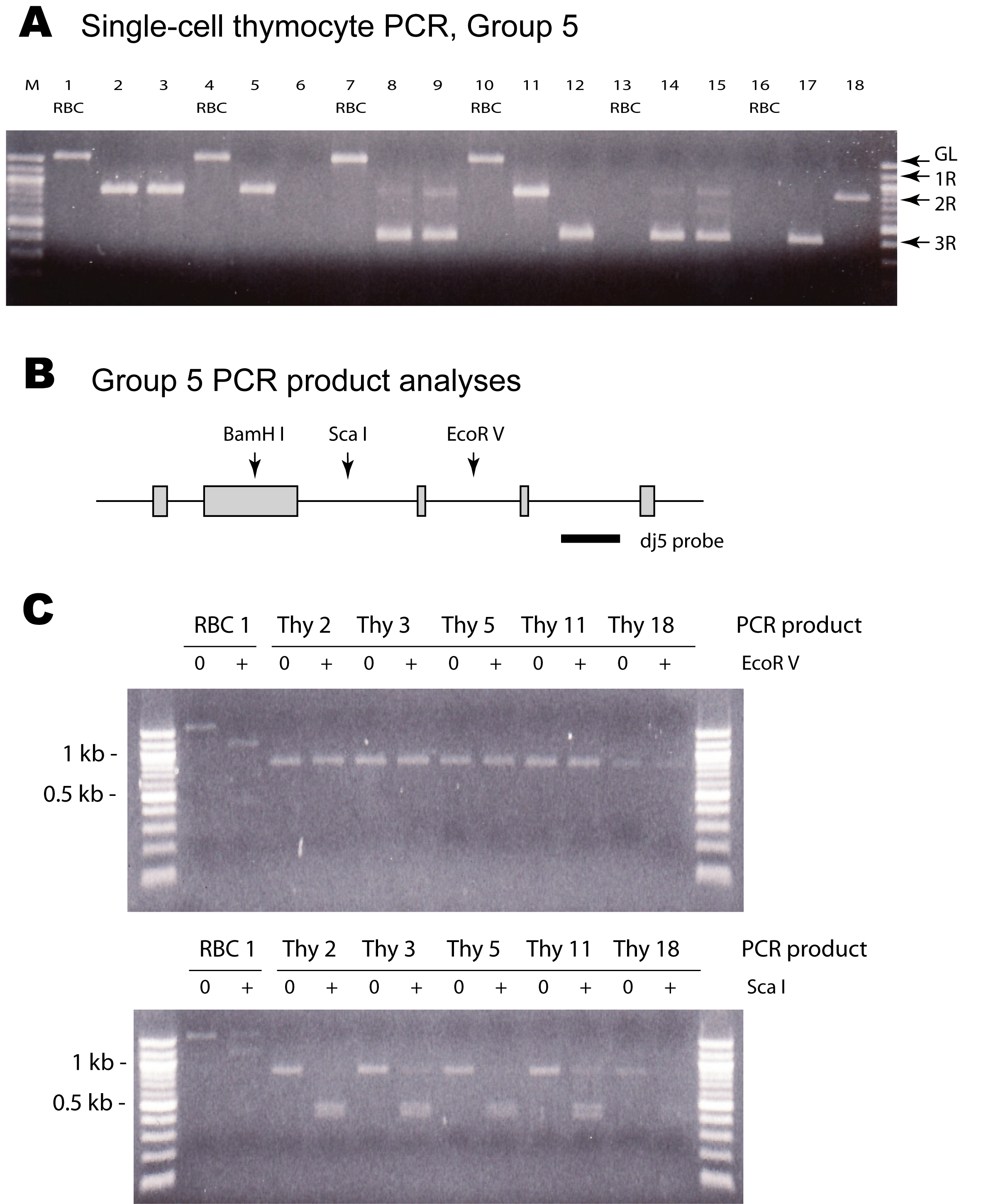

Supplement: Figure S7 — (A) The nested single-cell PCR was performed with a 5′ primer specific for Group 5 (FAM 5: 5′-GGCTCAGGATTCATTTCG-3′) and JH6. (B) The PCR products were characterized by use of probes to the Group 5 D-J region (dj5) and restriction enzyme analyses. (C) Restriction enzyme digests of PCR products from RBC control and thymocytes. Top, Sca I-digested samples; bottom, EcoRV. The PCR products are: Lane 1. RBC, GL (dj5 probe positive, BamHI+, ScaI+, and EcoRV+). Lane 2. thymocyte, 2R, V-DDJ (dj5 probe negative, BamHI+, and ScaI+). Lane 3. thymocyte, 2R, V-DDJ (BamHI+ and ScaI+). Lane 4. RBC, GL. Lane 5. thymocyte, 2R, V-DDJ (BamHI+ and ScaI+). Lane 6. dj5 negative. Lane 7. RBC, GL. Lane 8. thymocyte, 3R (BamHI+). Lane 9. thymocyte, 3R. Lane 10. RBC, GL. Lane 11. thymocyte, 2R, V-DDJ (BamHI+ and ScaI+). Lane 12. thymocyte, 3R. Lane 13. dj5 negative. Lane 14. thymocyte, 3R. Lane 15. thymocyte, 3R. Lane 16. dj5 negative. Lane 17. thymocyte, 3R. Lane 18. thymocyte, 2R, V-DDJ (BamHI+ and ScaI+). (5.53 MB TIF) [file pbio.0060157.sg007.tif]

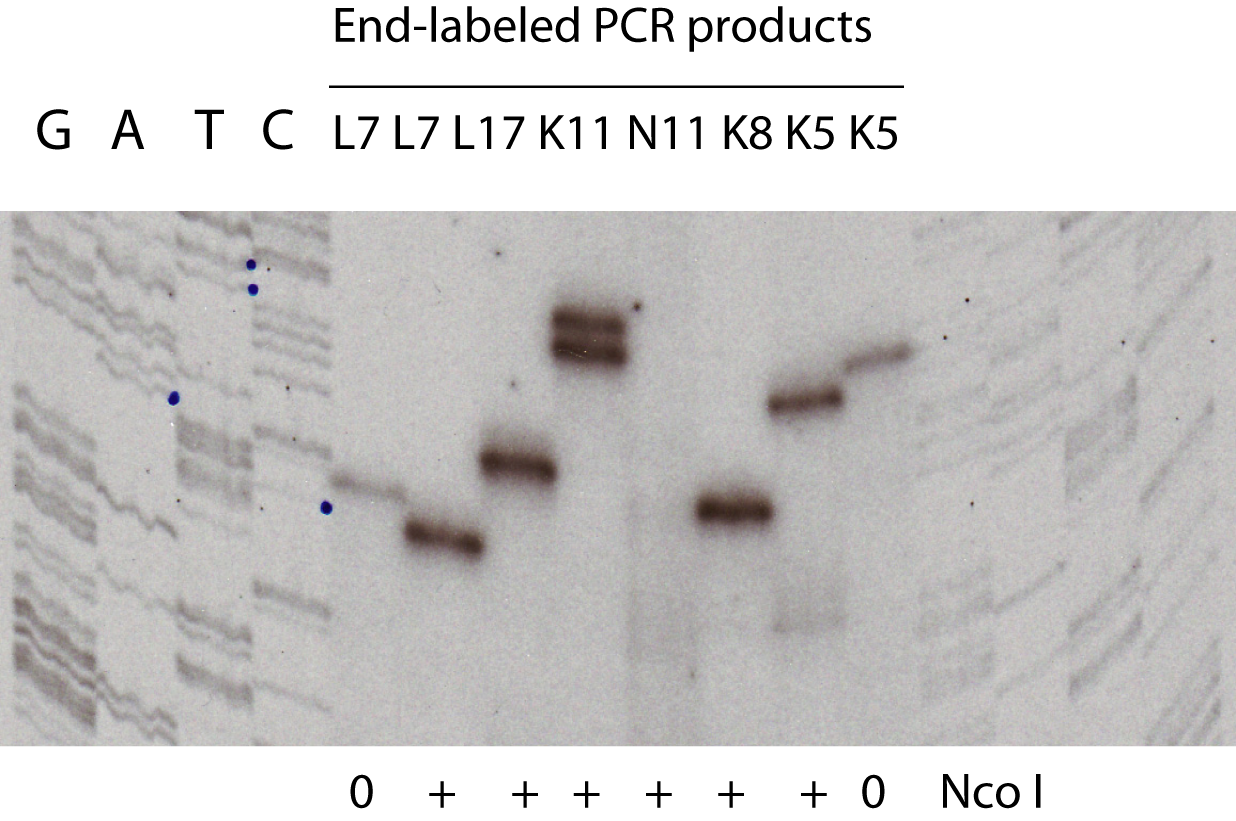

Supplement: Figure S8 — DNA samples from single-cell PCR of B cells were digested with NcoI, the recognition site being present in the JH6 primer that is used in the nested round of PCR (3R about 350–370 bp; see Figure 4, bottom). NcoI leaves a recessed 3′ end that can be filled in with 32P-CTP. The end-labeled samples were denatured and loaded on a sequencing gel along with the sequence of M13mp18 (lanes marked GATC). The blue dot at the bottom marks the C position at 339 bases of the phage, sequenced with the −40 primer (Sequenase Version 2.0, USB). There is some labeling in the absence of NcoI digestion (first and last lanes, L7 and K5). Lanes L7, L17, K8, and K5 show one band, whereas K11 show two (cloned as VDJ from Groups 4 and 5). Lane N11 was an artifact at 450 bp (determined by sequencing). The technique had been devised as a method of distinguishing Ig sequences utilizing the variability at CDR3 [45]. (1.54 MB TIF) [file pbio.0060157.sg008.tif]

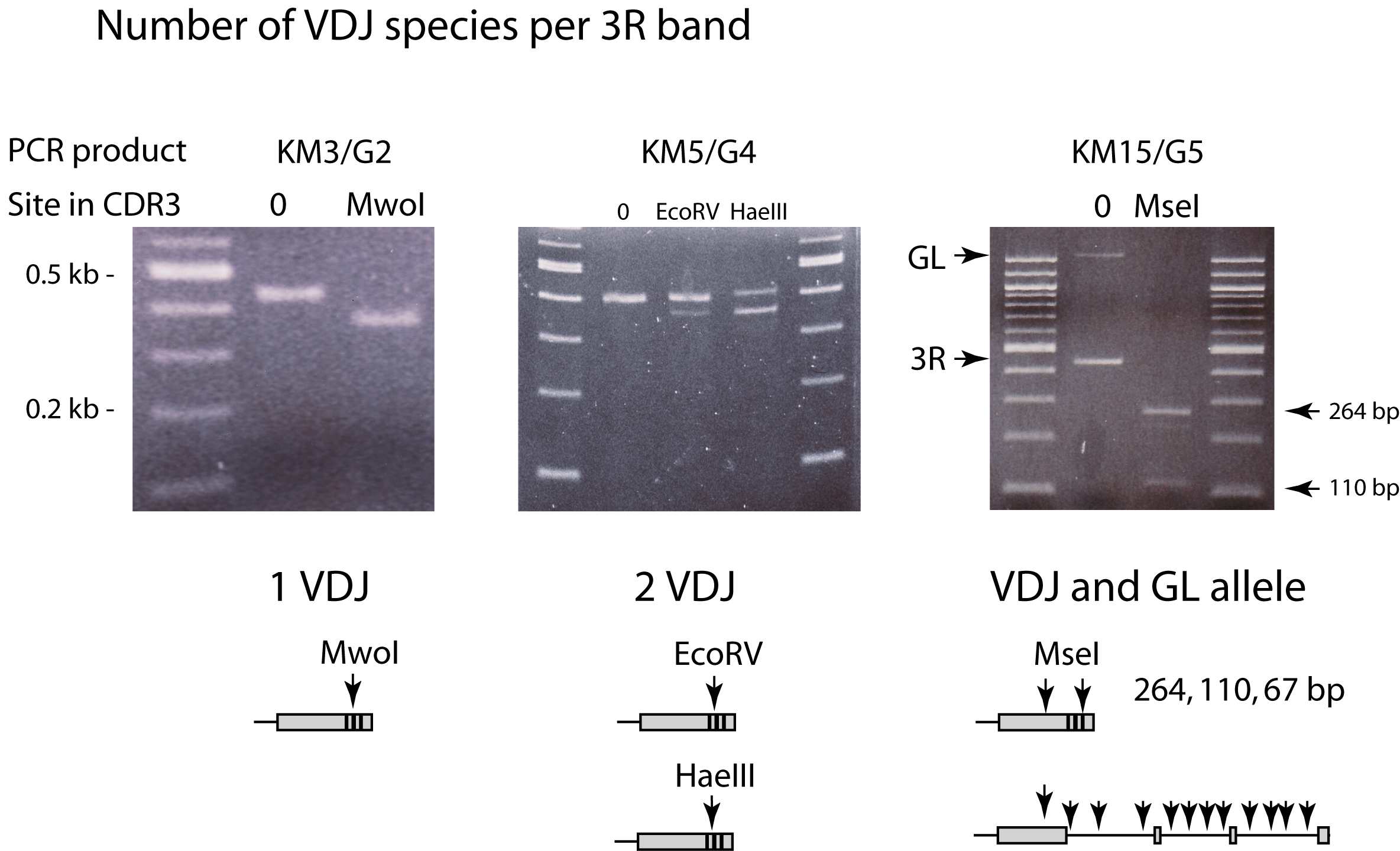

Supplement: Figure S9 — Every non-GL band found in the single-cell reactions (Figures 5–7) was cloned into pGEM vector after excising the band from an agarose gel and eluting the DNA using Qiagen columns. Usually the same sequence was obtained repeatedly in three to five clones. We determined whether there was one or more VDJ in the 3R band the following way. (1) Single bacterial colonies from the transformation would be suspended in LB medium and an aliquot subjected to PCR using the T7 and Sp6 primers to amplify the inserts. The 15–40 such PCR products would be digested with restriction enzymes, using a site in CDR3 that was found in the original sequence (listed in Figure S11). Every nondigested band was directly sequenced. (2) The original PCR product (both the universal primer product as in Figure 5 and the Group-specific product in Figures 6 and 7) would digested with the same restriction enzyme to ascertain whether all components of the band were digested. This is illustrated in the Panel KM3/G2 where the PCR product raised by Group 2-specific 5′ primer Int (and JH6) was completely digested with Mwo I. In the panel KM5/G4 the first sequenced plasmids contained a VDJ with an EcoRV site. The 3R fragment did not digest completely (as shown in lane 2), which suggested that a second, EcoRV-negative VDJ was present. Among 45 bacterial colonies, 18 carried an EcoRV site and 27 did not. The latter clones were grown up and sequenced, showing VDJ that carried a HaeIII site. Thus the cell sample KM5 carried two rearrangements of the G4 subfamily. Sometimes a GL sequence was amplified along with the 3R band as in panel KM15/G5. The VDJ carried two MseI sites, one in the VH and the second in CDR3, as diagrammed below the photograph of the gel. The 441-bp VDJ is expected to be digested by MseI into three fragments, 264 bp, 110 bp, and 67 bp. The 110-bp fragment is diagnostic and indicated with an arrow. There are multiple sites in the GL fragment, but these are present at a fraction of the VDJ an [file pbio.0060157.sg009.tif]

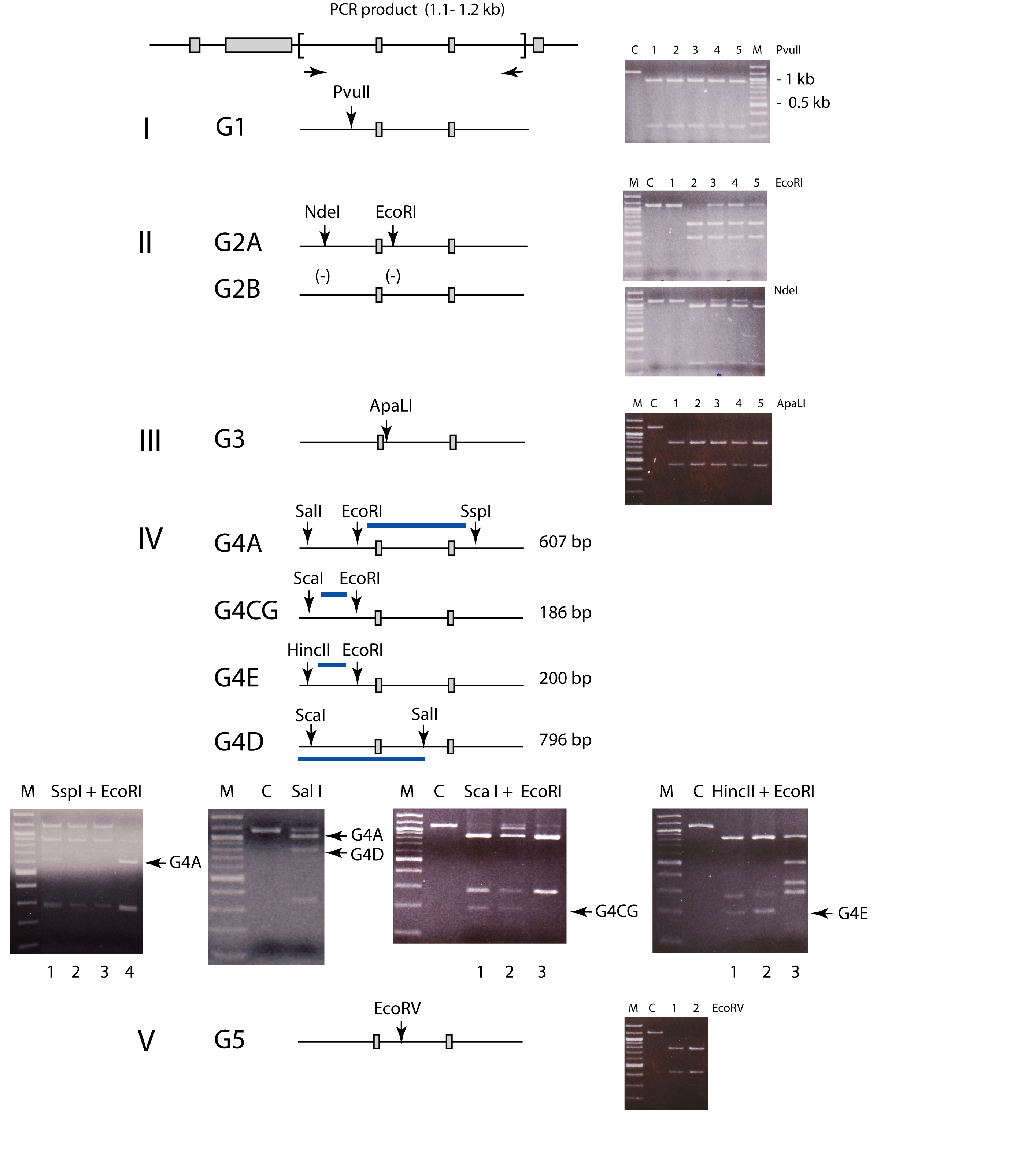

Supplement: Figure S10 — First-round PCR products from single B cells were subjected to nested PCR with primers in V-D and D-J that would amplify unrearranged sequence, bracketed in top diagram. The Group-specific primers separately amplified: Group 1 (1,226 bp), Group 2 (G2A, G2B: 1,154 bp), Group 3 (1,242 bp), Group 4 (G4A, G4D, G4E, G4C/G: 1,144–1,147 bp), and Group 5 (1,224 bp). I. Group 1 GL contains unique PvuII site in the V-D region, which can be detected as shown in agarose gel at right. GL sequences were amplified from single cells, which were confirmed to be G1 by presence of PvuII site. C is control, no enzyme. II. Group 2A GL sequence can be distinguished from Group 2B by two sites, NdeI and EcoRI, which are absent in G2B. In the gels, sample 1 carries G2B but not G2A; and sample 2 carries G2A but not G2B; the rest (3–5) carry both G2A and G2B. III. Group 3 GL contains an ApaLI site in the D-D region (as shown) but is negative for PvuII, EcoRI, and EcoRV (unpublished). IV. The four Group 4 GL sequences can be distinguished by a series of restriction enzyme digestions. A combination of SspI and EcoRI demonstrates whether G4A (607, 273, and 265 bp) are components of the PCR products. The bolded fragment, 607 bp, is indicated by the blue bar in panel IV, G4A, and is present only (arrows) in samples 1 and 4 in the first gel at left. In this photograph, the marker lane was transposed closer to the four sample lanes than it was in the original gel. To distinguish whether the G4 samples whose PCR products contain G4D or G4A or both, they were incubated with SalI (G4D: 796, 348 bp, G4A: 1019, 126 bp). The diagnostic band in the SalI gel are marked with arrows. C is control, no enzyme. The presence of G4CG and G4E involve somewhat more complex digestion patterns but the diagnostic bands are distinct. Digestions of the GL PCR product with ScaI and EcoRI provide only G4CG with a 186-bp fragment (G4CG: 874, 186, and 87 bp; G4A: 872 and 273 bp; G4D: 1,057 and 87 bp; and G4E: 874 and 273 bp) [file pbio.0060157.sg010.tif]

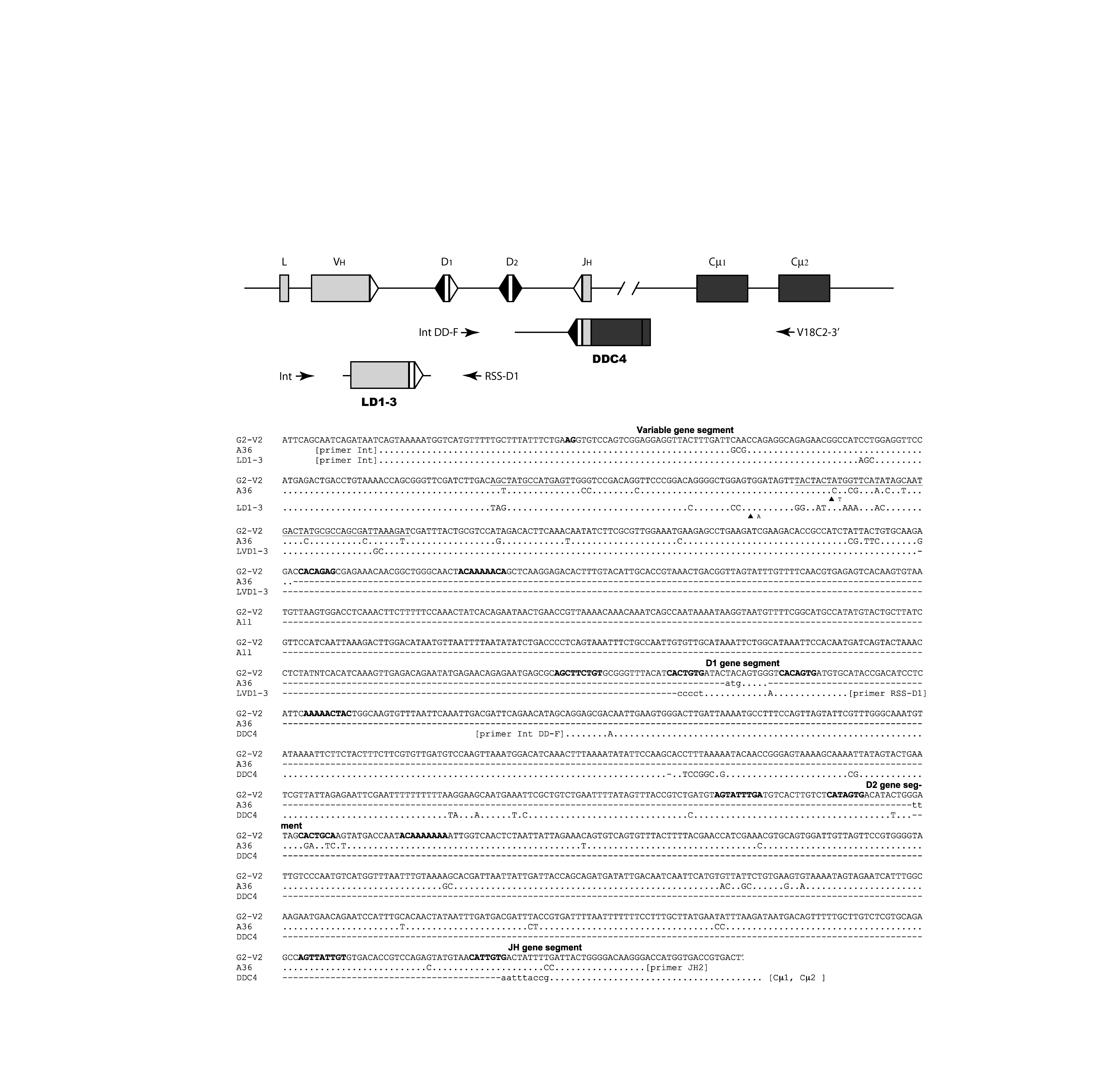

Supplement: Figure S12 — Top, diagram showing positions of two of the PCR primer pairs used and the transcripts detected relative to the GL genes. RT-PCR was performed on shark-JS spleen RNA with various PCR primer combinations described in Materials and Methods, and the products were cloned and sequenced. Bottom, representative mutated sequences are aligned to the GL gene G2-V2, whose VH, D1, D2, and JH gene segments are labeled. The CDR1 and CDR2 in the VH are underlined; RSS are bolded, as is the leader intron acceptor splice site. Clone A36: Int/JH2 primer pair, two-rearrangement VDD-J sequence (accession number: DQ857392). Clone LVD1–3: Int/RSSD1 primer pair, VD1 sequence, derived from VD-DJ or VD-D-J transcript. Clone DDC4: IntDD-F/V18C2–3′ primer pair, DJC sequence (accession number: DQ857391), derived from transcript carrying VD-DJ or V-D-DJ; its Group 2 C region sequence is not shown. These clones were chosen for the presence of mutations throughout the sequence; no portion was shared with any non-Group 2 sequences. The tandem substitutions are typical of hypermutated shark Ig [26]. Dots denote identity with the reference sequence, dashes gaps. Substitutions are shown in capital letters, N region in lower case in front of the D or JH sequence. Insertions are marked with arrow. Sites of the PCR primers are indicated in brackets. (856 KB TIF) [file pbio.0060157.sg012.tif]

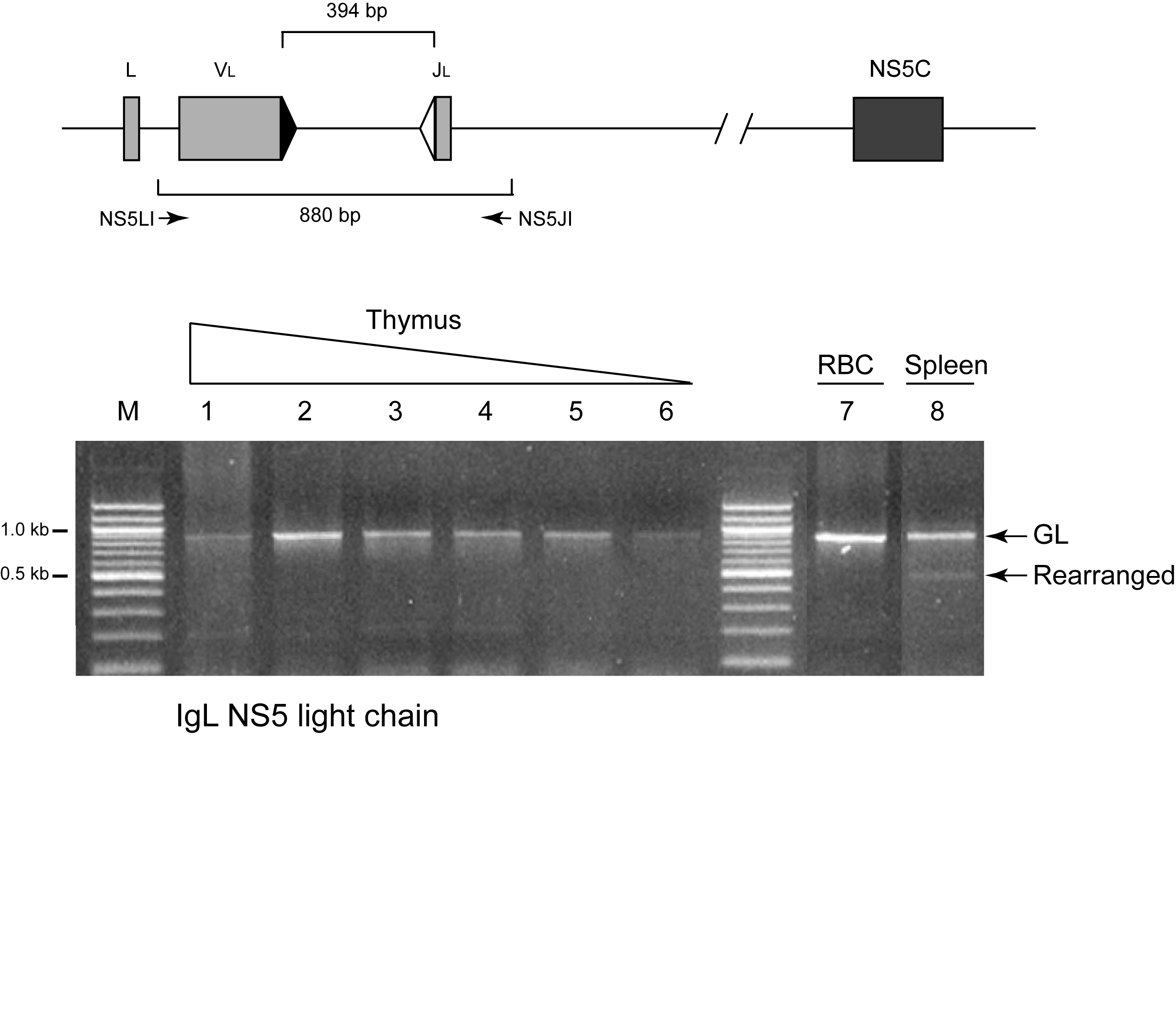

Supplement: Figure S13 — Diagram at top: organization of the NS5–2 L chain locus and location of the primers. Boxes represent coding sequences of leader, V and J gene segments and C exon, filled triangle an RSS with 12-bp spacer, open triangle an RSS with 23-bp spacer. Bottom: 1.5% agarose gel, PCR was performed using primers in the leader intron and 3′ of JL, that detect the NS5–2 L chain gene [29], GL V-J (arrow, GL at 880 bp) and rearranged VJ (∼490 bp), in spleen DNA (lane 8). The RBC lane was inserted from another position (11th lane) on the same gel. (1.19 MB TIF) [file pbio.0060157.sg013.tif]
