# Supplementary material for: Immunoglobulin Heavy Chain Exclusion in the Shark
Source: PLoS Biol. 2008 Jun 24;6(6):e157. doi: 10.1371/journal.pbio.0060157 (PMC2435157; doi:10.1371/journal.pbio.0060157)
Supplement: Figure S11 — The CDR3 of rearranged VDJ from single B cells shown in Table 3 are aligned under the GL flanks of the VH and the JH gene segments and the coding regions of D1 and D2. The trimmed positions are shown with dashes, for gaps, and other sequences are assigned as N or P nucleotides. The GL sequences were cloned from shark-GR, so that mutated positions could be identified clearly (indicated as lower case). “+” indicates in-frame potentially functional sequence, “non” is in-frame CDR3 with stops (underlined), null symbol indicates out-of-frame sequence. The VDD-J in KS23 has undergone only two rearrangement events. The CDR3-based restriction enzymes listed at the right were used to detect the number of VDJ species per 3R band. (36 KB DOC) [file pbio.0060157.sg011.doc]

VH flank N/P D1 N/P D2 N/P JH flank CDR3

**KM1** site

Gr.2B TGTGCAAGAGAC ATACTACAGTGGGT ACATACTGGGATAG ACTATTTTGGTTACTGG

KM1+ TGTGCAAGA--- TCTCGCC ACTAC GC CATACTGGGAT CCTACTCTTT ----TTTTGGTTACTGG BglII

# KM3

Gr.2A TGTGCAAGAGAC ATACTACAGTGGGT ACATACTGGGATAG ACTATTTTGATTACTGG

KM3ø TGTGCAAGA--- TACG CTACAGTGGG GTCGGATGTATAG GGGATA CTGGGACGATA --------GATTACTGG MwoI

# KM5

Gr.4G/C TGTGCAAGAGAC ATACTACAGTGGGT ACATACTGGGGTAG ACGAATTCGCTAACTGG

KM5+ TGTGCAAGAGAC CAGG ATAtcACAG CCC GTAG ACTCTCA -----TTCGCTAACTGG EcoRV

KM5ø TGTGCAAGA--- ACA CGGGGCCGGAT GGGT CA ------TCGCTAACTGG HaeIII

# KM13

Gr.1 TGTGCAAAAAGC AGTGGGT ATACACTGGATTGG GCTATCTTGATCACTGG

KM13+ TGTGCAAA---- GCACAGACA TGG AAG CTGG CGAGGTG ------TTGATCAgTGG MslI

Gr.2A TGTGCAAGAGAC ATACTACAGTGGGT ACATACTGGGATAG ACTATTTTGATTACTGG

KM13ø TGTGCAAG---- GCCCCTAT ATACTACAGTG CG ACTGGG GGG -----TTTGATTACTGG SfcI

Gr.4G/C TGTGCAAGAGAC ATACTACAGTGGGT ACATACTGGGGTAG ACGAATTCGCTAACTGG

KM13+ TGTGCAAG---- CG AGTGGGT ATTCGGTCGGG GGGGT TTCCGTTCTC ----ATTCGCTAACTGG SmaI

ATTTTCCC TCACTCCGCA

TGCCGGGGCG

# KM15

Gr.5 TGTGCAACAGAT ATACTACAGTGGGT ACATACTTGGGTGG ACTACTCCGGTTACTGG

KM15non TGTGCAA----- ACGGGGGTTAA ACAGTG AACTTGATC ACTTGGGTG TCCAGCGG ----CTCCGGTTACTGG MseI

**KM17**

Gr.4D TGTGCAAGAGAC ATACTACAGTGGGT ACATACTGGGGTAG ACGAATTCGGTAACTGG

KM17+ TGTGCAAG---- CAG ACAG GAC TAC G ------------ACTGG BstNI

**KM33**

Gr.4G/C TGTGCAAGAGAC ATACTACAGTGGGT ACATACTGGGGTAG ACGAATTCGCTAACTGG

KM33+ TGTGCA------ TGGAGCCT CAGTGG TCGG CAT CCCAGC --------GCTAACTGG HaeII

**KS1**

Gr.4G/C TGTGCAAGAGAC ATACTACAGTGGGT ACATACTGGGGTAG ACGAATTCGCTAACTGG

KS1+ TGTGTtAGAGA- T TACT CATcCT ACGCC -----TTCGCTAACTGG sequenced*

**KS3**

Gr.2A TGTGCAAGAGAC ATACTACAGTGGGT ACATACTGGGATAG ACTATTTTGATTACTGG

KS3non TGTGCAAG---- T CAG CCCCGGT GATA A -----------TACTGG HpaII

Gr.4D TGTGCAAGAGAC ATACTACAGTGGGT ACATACTGGGGTAG ACGAATTCGGTAACTGG

KS3ø TGTGCAAGA--- TAGGCCG ACAGTGG CCCATCGGCGGGG ACTGGG CCATAT ACGAATTCGGTAACTGG MwoI

Gr.5 TGTGCAACAGAT ATACTACAGTGGGT ACATACTTGGGTGG ACTACTCCGGTTACTGG

KS3non TG---------- GGTATGC CTACAGTG AGATTCGG GGGTGG TGGGATTCGGG --------GGTTACTGG DdeI

TCAGACGGCAT

ACGATCTGAGCACTCC

# KS11

Gr.2A TGTGCAAGAGAC ATACTACAGTGGGT ACATACTGGGATAG ACTATTTTGATTACTGG

KS11ø TGTGCAAG---- TGGGGCCGTGA ACAGTGG TAC CGCCCCGATC ---ATTTTGATTACTGG KpnI

CGG TCCCC

**KS23**

Gr.2A TGTGCAAGAGAC ATACTACAGTGGGT ACATACTGGGATAG ACTATTTTGATTACTGG

KS23V2+ TGTGCAAGAGAC GGAC TACTACA CTTCG GGGA CCG -----TTTGATTACTGG sequenced*

Gr.2A TGTGCAAGAGAC ATACTACAGTGGGT ACATACTGGGATAG ACTATTTTGATTACTGG

KS23V1n TaTGCAAG---- TGGGGG AGT TGGGATAG GL sequenced*

Gr.4A TGTGCAAGAGAC ATACTACAGTAGGT ACATACTGGGGGAG ACTACTTTGATAACTGG

# KS23+ TGTGCAAG---- CTC CAG ACG CATAC CGGATCGT ----------TAACTGG HincII

**KS51**

Gr.4G/C TGTGCAAGAGAC ATACTACAGTGGGT ACATACTGGGGTAG ACGAATTCGCTAACTGG

KS51+ TGTGCAAGAG-- GGGAG CTACAGTGGGT GCGGGAAA ---AATTCGCTAACTGG SfcI

**KS53**

Gr.4G/C TGTGCAAGAGAC ATACTACAGTGGGT ACATACTGGGGTAG ACGAATTCGCTAACTGG

KS53ø TGTGCAAGA--- GCCCGATT CAGT CTCACCT CAT CG -----TTCGCTAACTGG HaeIII

*The PCR 3R product was electrophoresed on agarose gel, excised, and isolated by Qiagen columns. The DNA thus

obtained was sequenced in both directions and determined to consist of one species by the electropherogram output.
