# Supplementary material for: Immunoglobulin Heavy Chain Exclusion in the Shark
Source: PLoS Biol. 2008 Jun 24;6(6):e157. doi: 10.1371/journal.pbio.0060157 (PMC2435157; doi:10.1371/journal.pbio.0060157)
Supplement: Text S1 — (82 KB DOC) [file pbio.0060157.sd001.doc]

**Glossary of terms**

**B cells, T cells** are the two subsets of lymphocytes that provide the basis of the adaptive immune response through their antigen receptors. The mature lymphocyte circulates in the **blood**, lymph, and through secondary lymphoid tissues such as **spleen**, lymph nodes, and Peyer’s Patches. Each mature B cell expresses one kind of immunoglobulin (Ig) receptor that is a four-polypeptide unit of two identical heavy (H) chains and two identical light (L) chains. The H chain C-terminus is encoded by constant (C) region genes that give the Ig its classification, IgM. The N-terminus provides the antibody combining site (V region) that was generated by V(D)J rearrangement (see Figure 1). By definition a cell expressing IgM on its surface (sIgM+) is a B lymphocyte. After stimulation by antigen in secondary lymphoid organs, B cells develop into antibody-secreting cells. T cells express only T cell receptor (TCR). Only **T and B lymphocytes** have their antigen receptor genes rearranged.

**Blood** contains erythrocytes (red blood cells, **RBC**) and non erythrocytes (**peripheral blood leukocytes, PBL**). The two populations can be separated by centrifuging whole blood through a density gradient (Ficoll). Since the shark RBC are nucleated and are present at a ratio of 250 to 1 PBL, the majority of genomic DNA from whole blood will be from erythrocytes.

**PBL:** the “white cell” component in blood, consists of T cells, B cells, granulocytes, thrombocytes, and monocytes. Lymphocytes tend to be a minor population in the PBL and the proportion may fluctuate according to the immune status of the individual.

**Epigonal organ** is the nurse shark **bone marrow** equivalent. Bone marrow contains pluripotent hematopoietic stem cells that give rise to cells circulating in the blood. It is the site of B lymphocyte development. **Precursor B cells** rearrange their Ig genes in the bone marrow, and after they successfully express a functional antigen receptor (sIgM) they exit and enter the circulation as mature B lymphocytes. B cells that are actively rearranging their Ig genes are restricted to the bone marrow. Incomplete or out-of-frame rearrangements in a mature B cell are relicts from this earlier stage of differentiation.

**Spleen:** Secondary lymphoid organ that filters blood and traps potential antigens. T and B cells passage through it and can constitute ~90% of the non-RBC population. Other cells mostly consist of macrophages, other leukocytes, and epithelial cells.

**Thymus** is an organ consisting of a network of epithelia enclosing T cell precursors and is the site of T lymphocyte development. Progenitor cells exit the bone marrow and enter the thymus. **Precursor T cells** undergo rearrangement of T cell receptor (TCR) genes in the thymus and exit into circulation as mature T cells. T cells of various stages of differentiation constitute the vast majority of thymic cells, and other non-lymphocyte cells include epithelial cells, dendritic cells, nurse cells and macrophages. Isolated **thymocytes** are immature T cells that are in the process or have rearranged their TCR genes.
